# New mechanistic insights into macrophage extracellular trap formation induced by a parasitic nematode, *Strongyloides stercoralis*

**DOI:** 10.3389/fimmu.2025.1636232

**Published:** 2025-10-24

**Authors:** Taoxun Zhou, Bingying Zhang, Runxin Zhu, Chunqun Wang, Hui Liu, Nishith Gupta, Min Hu

**Affiliations:** ^1^ National Key Laboratory of Agricultural Microbiology, College of Veterinary Medicine, Huazhong Agricultural University, Wuhan, China; ^2^ Intracellular Parasite Education and Research Labs (iPEARL), Department of Biological Sciences, Birla Institute of Technology and Science, Pilani (BITS-Pilani), Hyderabad, India; ^3^ Department of Molecular Parasitology, Faculty of Life Sciences, Humboldt University, Berlin, Germany

**Keywords:** extracellular traps, macrophage, nuclear envelope, phosphoproteomics, Strongyloides stercoralis

## Abstract

Macrophages execute host defense against pathogens by releasing extracellular traps (METs) composed of DNA meshwork and antimicrobial proteins. Although MET-mediated pathogen immobilization is well documented, the induction mechanisms of MET generation by helminth parasites remain elusive. Here, we demonstrate that *Strongyloides stercoralis* larvae induce rapid chromatin extrusion in murine macrophages. Unlike neutrophil extracellular trap (NET) formation, MET formation does not require NADPH oxidase and exhibits distinct ultrastructural characteristics, including endoplasmic reticulum vesiculation, perinuclear space dilation, and inner nuclear membrane budding. Phosphoproteomic analysis revealed that MET formation is coordinately regulated by ERK and AKT signaling, F-actin cytoskeletal remodeling, histone acetylation, and phosphorylation of nuclear envelope (NE) proteins. Specifically, we show that protein kinase C zeta isoform (PKCζ)-mediated lamin A/C phosphorylation drives the NE budding and subsequent DNA expulsion. This work represents the first systematic delineation of the cellular dynamics and molecular machinery underlying MET formation, providing new insights into macrophage-directed anti-helminth immunity.

## 1 Introduction

Soil-transmitted helminths threaten one-quarter of the global population (1). Among these pathogens, *Strongyloides stercoralis*, the primary causative agent of strongyloidiasis, remains a critically neglected tropical disease despite causing an estimated 600 million global infections, representing a persistent public health challenge (2). Infection initiates when infective third-stage larvae (iL3) penetrate the skin, subsequently migrating via the blood-pulmonary route to the small intestine. Within duodenal crypt mucosa, larvae mature into parthenogenetic parasitic females that release eggs, hatching into post-parasitic first-stage larvae (PPL1) (3, 4). A subset of PPL1 develops rapidly into auto-infective third-stage larvae (aL3), perpetuating infection through intestinal wall/perianal skin reinvasion before fecal excretion (5, 6). This autoinfection cycle drives persistent parasitism, culminating in lethal disseminated hyperinfection in immunocompromised individuals (7). However, the lack of effective vaccines underscores the imperative to decipher the molecular mechanisms governing the host protective immunity against *S. stercoralis*.

Although immunocompetent mice resist patent infections of *S. stercoralis* (8), the experimental challenge infection with iL3 enables the investigation of host early immune responses against the initial phase of infection (9), significantly advancing our mechanistic understanding of anti-larvae immunity (10). Notably, the oral transfer of parasitic females enables parasite colonization in the intestine and results in progeny production in the murine model (11), indicating that immunity targeting the larval migratory phase is critical for host resistance against *S. stercoralis*. During larval migration through tissues, innate immune cells, primarily neutrophils, eosinophils, and macrophages, are recruited to larval microenvironments (12). Larval killing by granulocytes is mediated by their respective granule proteins—myeloperoxidase (MPO) in neutrophils and major basic protein (MBP) in eosinophils (13). By contrast, the immune strategies employed by macrophages against *S. stercoralis* infection remain underexplored.

Macrophages are versatile cells involved in immune defense, tissue repair, and homeostasis while contributing to immunopathology (14, 15). Alternatively activated macrophages (AAMs) play a crucial role in type 2 anti-helminth immunity, contributing to helminth clearance and tissue repair (16, 17). This functional repertoire extends to *S. stercoralis* clearance, where macrophages cooperate with neutrophils to kill iL3, with AAMs exhibiting enhanced larvicidal activity during both primary and secondary infections (18). However, the macrophage-mediated larvicidal effect requires direct contact with larvae (18). The striking migratory disparity between iL3 (10 cm/h tissue penetration rate) and host immune cells (0.06 cm/h migratory rate) creates a spatiotemporal paradox for effector cell-parasite contact (10). Thus, conventional experimental approaches—including *in vitro* co-culture systems or subcutaneous diffusion chamber models that physically constrain larval mobility—fail to capture the spatiotemporal coordination required for macrophages to intercept rapidly migrating larvae *in vivo*.

Extracellular traps (ETs) are web-like structures composed of decondensed chromatin decorated with antimicrobial proteins, representing a conserved defense mechanism employed by innate immune cells to ensnare and eliminate pathogens (19). Emerging evidence establishes ETs released by neutrophils and eosinophils as pivotal effectors against helminth infections, including nematodes (20–24) and platyhelminths (25–28). ETs immobilize and/or kill helminth parasites (29), providing an evolutionarily conserved strategy to counteract pathogens exceeding phagocytic capacity. In contrast to the well-documented induction of macrophage extracellular traps (METs) by protozoan parasites (30–32), helminth-induced MET formation remains an uncharted frontier in innate immunology. A recent study identified *Trichinella* sp*iralis*-induced METs with helminthicidal activity (33), contradicting earlier reports that mouse macrophages lack MET-generating capacity against *S. stercoralis* (34). Given the enhanced larval clearance observed in murine models, the ability of mouse macrophages to release METs against *S. stercoralis* and the underlying mechanisms remain to be established.

Therefore, the current study investigates the capability of mouse macrophages to produce METs in response to *S. stercoralis* iL3 and elucidates the molecular mechanisms of MET formation. Our findings provide novel insight into the role of macrophage-specific anti-helminth immunity.

## 2 Materials and methods

### 2.1 Parasites and animals

Immunocompromised beagles were infected with *S. stercoralis* UPD (University of Pennsylvania Dog strain). Larvae were collected according to a standard procedure described previously (5). Dog feces were collected, mixed with charcoal, and cultured in a moist incubator at 22 °C. The infective third-stage larvae (iL3) were collected following culture for 7 days using the Baermann funnel technique (5). Worms were sterilized with 2 mM sodium hypochlorite for 5 min, thoroughly washed with phosphate-buffered saline (PBS), and resuspended in a serum-free culture medium. Decontamination of the larvae was determined by aerobic culture.

Female 6-8-week-old C57BL/6 mice were housed in a standard specific pathogen-free (SPF) animal facility, at a temperature of 24 °C and a humidity-controlled environment with 12 h day-night cycles, and provided with water and food *ad libitum* in the Laboratory Animal Center of Huazhong Agricultural University. Mice were sacrificed by CO_2_ asphyxiation and cervical dislocation.

### 2.2 Cells and bacteria

RAW264.7 and HEK293T cell lines were grown and maintained in DMEM supplemented with 10% fetal bovine serum (FBS, Gibco), 2 mM L-glutamine and 100 I.U./mL penicillin–streptomycin in tissue culture dishes or flasks at 37 °C, 5% CO_2_. *Mycoplasma* contamination was tested before experiments.

Peritoneal macrophages were harvested as described elsewhere (35) with some modifications. Briefly, resident macrophages were collected by peritoneal lavage with cold PBS containing 10 mM EDTA, and centrifugation at 100 g for 10 min. Cells were resuspended in RPMI 1640 supplemented with 2% heat-inactivated FBS and then cultured at 37 °C, 5% CO_2_ for 3 h. Nonadherent cells were removed by repeatedly shaking and discarding supernatants. Adherent macrophages were scraped off, counted, and seeded in plates for MET induction.

*Mycobacterium smegmatis* (MC^2^ 155 strain) was cultured in Middlebrook 7H9 broth medium supplemented with OADC (oleic acid, albumin, dextrose, catalase), 0.2% glycerol, and 0.05% Tween 80. Middlebrook 7H10 agar plates were used for bacterial colony counting.

*Escherichia coli* was cultured in an LB medium*. E. coli* DH5α was used for standard cloning and vector construction. Lentiviral plasmids were maintained in *E. coli* Stbl3.

### 2.3 MET induction and DNA quantification

1.25 × 10^5^ cells were seeded in 24-well plates with 2% FBS overnight. The culture medium was removed and replaced with a fresh medium without serum, phenol red, and antibiotics. After incubation for 2 h, cells were exposed to sterilized worms or other stimuli for the indicated time. Cell supernatants were collected and centrifuged at 3,000 *g* for 5 min to remove cell debris and worms. DNA concentration was measured using Quant-iT PicoGreen™ dsDNA Kit (Invitrogen) following the manufacturer’s instructions. Briefly, cell supernatants in 96-well black microplates were mixed with picogreen reagent working solution (1:1) and incubated at room temperature (RT) for 5 min. The samples were excited at 480 nm and the fluorescence emission intensity was measured at 520 nm using a microplate reader (Bio Tek). A DNA standard curve was generated for each detection to calculate the DNA concentration of the samples.

For pharmacological inhibition, chemical drugs were added to the culture medium 30 min before treatment with worms. Inhibitors/chelators for NADPH oxidase (diphenyleneiodonium chloride, DPI; Selleck), MPO (4-Aminobenzohydrazide, Selleck), neutrophil elastase (Ac-YVAD-cmk, Selleck), ROS (N-acetylcysteine, Selleck), Ca^2+^ (EGTA, Macklin; BAPTA-AM, Selleck), microfilament (Cytochalasin D, Invitrogen), RNA polymerase II (Actinomycin D, Selleck), HDACs (Panobinostat, Selleck) and AKT (MK-2206, Selleck). Pamoic acid (Selleck) was used as an ERK agonist.

### 2.4 Immunofluorescence assay

1.25 × 10^5^ cells suspended in culture medium with 2% FBS were seeded on 14 mm poly-L-Lysine-pretreated coverslips in 24-well plates overnight. After MET induction described above, coverslips were fixed with 4% paraformaldehyde solution and permeabilized with 0.1% Triton X-100 for 15 min, followed by blocking for 1 h in 2% w/v BSA, 22.52 mg/mL glycine in PBST (PBS with 0.1% v/v Tween-20) at RT. Primary antibody (Myeloperoxidase, Abcam; Histone 3, Abclonal) incubation was performed overnight at 4°C or for 2 h at RT in a moist chamber with primary antibodies diluted in 2% w/v BSA in PBST supplemented with 0.1% v/v microbicide ProClean 150 (Beyotime). The primary antibodies were washed off and sections were incubated with Alexa Fluor™ 594 goat anti-rabbit secondary antibodies (Invitrogen) for 1 h at RT. Following washing, the coverslips were counterstained with 5 μg/ml Hoechst 33258 (Beyotime) at RT for 10 min. Finally, coverslips were mounted in Antifade Mounting Medium (Beyotime).

Fluorescence microscopy was performed using an Olympus biological microscope (BX53) with a × 40 and a × 100 objective. Confocal microscopy was performed using a Zeiss LSM 800 confocal laser scanning microscope with an airyscan detector. Fluorescence images were edited and processed using ZEISS ZEN software (https://www.zeiss.com/microscopy/en/products/software/zeiss-zen.html).

### 2.5 Lactate dehydrogenase release assay

Cell supernatants were collected and centrifuged at 3,000 *g* for 5 min at 4 °C. Samples were incubated with a working solution for 30 min at RT, according to the manufacturer’s instructions of the LDH Release Assay Kit (Beyotime). Absorbance was measured at 490 nm.

### 2.6 PCR and quantitative real-time PCR

Total RNA was extracted using TransZol (Transgen), and the first strand cDNA was synthesized using HiScript III RT SuperMix reverse transcription Kit (Vazyme). Real-time PCR was performed using the SYBR qPCR Kit (Vazyme). ΔCt values were normalized to *β-Actin*, and relative quantification of gene expression was compared to the control group without actinomycin D (Selleck) treatment.

Nuclear/mitochondrial DNA (nDNA/mtDNA) determination was performed as previously described (24) with modifications. First, extracellular DNA was purified from supernatants of macrophages without stimulation (control) or with iL3 stimulation using EasyPure Genomic DNA Kit (Transgen). Then, PCR followed by agarose gel electrophoresis was conducted to detect nuclear DNA and mitochondrial DNA fragments in the purified supernatant DNA. Finally, qPCR was performed using SYBR qPCR Kit to amplify nuclear genes (actin beta (*Actb*), glycerinaldehyd-3-phosphat-dehydrogenase (*Gapdh*) and mitochondrial genes (NADH-ubiquinone oxidoreductase chain 1 (*Nd1*), ATP synthase membrane subunit 6 (*Atp6*)). nDNA/mtDNA fold change was calculated as follows: Control ΔCt = Ct (nDNA) - Ct (mtDNA) in the control group; iL3 ΔCt = Ct (nDNA) – Ct (mtDNA) in the iL3-treated group; ΔΔCt =iL3 ΔCt – average Control ΔCt; nDNA/mtDNA fold change = 2^-ΔΔCt^. Primers used are listed in the reagents and tools table.

The primer pairs were used as follows: *Tnf-a*, forward primer 5’-TTCTCATTCCTGCTTGTGGCA-3’ and reverse primer 5’-TGATGAGAGGGAGGCCATTTG-3’; *β-Actin*, forward primer 5’-GCTCAGTAACAGTCCGCCTAGAA-3’ and reverse primer 5’-ATCCTTAGCTTGGTGAGGGTG-3’; *Atp6*, forward primer 5’-AGGATTCCCAATCGTTGTAGCC-3’ and reverse primer 5’-CCTTTTGGTGTGTGGATTAGCA-3’; *Nd1*, forward primer 5’-TCACTATTCGGAGCTTTACGAGC and reverse primer 5’-CATATTATGGCTATGGGTCAGGC-3’; *Gapdh*, forward primer 5’-ATGGCCTTCCGTGTTCCTAC and forward primer 5’- GGAGTTGCTGTTGAAGTCGC-3’.

### 2.7 Expansion microscopy

The ExM procedure was conducted based on the protocol described previously (36). Briefly, coverslips were fixed, permeabilized, and blocked as described above. Then, coverslips were immersed in FA/AA mix and incubated at 37°C for 5 h. Mix monomer solution with TEMED and APS through a quick vortex and immediately place approximately 40 μL per coverslip on the parafilm on ice. For gel polymerization, coverslips were mounted on the liquid drops for 5 min and then transferred to a 37°C incubator for 1 h. Next, coverslips were soaked in a denaturization buffer for 15 min with gentle agitation to detach the gels from the coverslips. Gels were then moved into tubes in fresh denaturation buffer and incubated at 95°C for 30 min. Gels were expanded in 100 mL beakers filled with about 50 mL ddH_2_O for 30 min repeatedly for 3 times by exchanging the water with the same volume and re-incubation.

After overnight expansion in ddH_2_O, gels were stained in 10 μg/mL Hoechst solution in ddH_2_O for 5h. Gels were washed with ddH_2_O 3 times, with 30 min each time. Finally, the gels were cut, and their central parts were mounted on the poly-L-lysine-pretreated glass-bottom dishes for confocal microscopy.

### 2.8 Transmission electron microscopy imaging

After exposure to larvae for the indicated time, cells were washed and fixed, followed by scraping off and centrifugation. Cell precipitates were preserved in fresh 2.5% glutaraldehyde solution at 4°C overnight. After washing with 0.1 M PBS 3 times, post-fixation was performed using 1% osmium tetroxide solution for 3 h. Samples were dehydrated by acetone solution (30%-50%-70%-80%-90%-100%-100%-100%). Resin components [SPI-Pon™ 812 Resin, (2-Dodecen-1-yl) succinic Anhydride, Methyl-5-norbornene-2,3- dicarboxylic Anhydride (12:1:3)] were thoroughly mixed for 12 h. Samples were infiltrated with acetone: resin (5:1-3:1-1:1-1:3-1:5) followed by complete resin. 1.5-2% 2,4,6-tris (Dimethylaminomethyl)-phenol was added to a resin and stirred for 12 h to generate the embedding solution. Samples were embedded in capsules with embedding solution and cured in a 60°C oven for 48 h.

Next, ultrathin sections were produced using Ultramicrotome (Leica UC6), loaded on nickel grids, and contrasted with saturated uranyl acetate solution for 30 min. Images were captured using 120 kV transmission electron microscopy (HITACHI H-7650/HT7800).

### 2.9 Cell viability assay

Cells were seeded in 96-well plates in a culture medium supplemented with 2% FBS overnight. Cells were exposed to chemical inhibitors in a serum-free medium for 3 h at different concentrations followed by incubation with Cell Counting Kit-8 (CCK-8) (Abbkine) reagent (10 µL per well) for an additional 1 h. Absorbance was measured at 450 nm.

### 2.10 Sample preparation for quantitative phosphoproteomics

RAW264.7 cells were exposed to iL3 or not for 30 min and scraped off on ice. Samples were sonicated three times on ice using a high-intensity ultrasonic processor (Scientz) in lysis buffer with 8 M urea, 1% protease inhibitor cocktail, and 1% phosphatase inhibitor cocktail. The debris was removed by centrifugation at 12,000 *g* at 4°C for 10 min. Next, the supernatant was collected, and the protein concentration was determined using the BCA kit (Beyotime) according to the manufacturer’s instructions. For digestion, the lysates were reduced with 5 mM dithiothreitol for 30 min at 56°C and alkylated with 11 mM iodoacetamide (Sigma-Aldrich) for 15 min at RT in darkness. The protein sample was then diluted by adding 100 mM Tetraethylammonium bromide (TEAB) (Sigma-Aldrich) to urea (Sigma-Aldrich) concentration less than 2 M. Trypsin was added at a 1:50 trypsin-to-protein mass ratio for the first digestion overnight and 1:100 trypsin-to-protein mass ratio for a second 4 h-digestion. Finally, the peptides were desalted by the C18 solid‐phase extraction (SPE) column.

### 2.11 Tandem mass tag based liquid chromatography-tandem mass spectrometry

The TMT labeling quantitative proteomics and phosphoproteomics analysis was performed by Jingjie PTM BioLab Co. Ltd (China). Tryptic peptides were first dissolved in 0.5 M TEAB. Each channel of peptide was labeled with its respective TMT labeling reagent based on the manufacturer’s introduction (ThermoFisher Scientific), and incubated for 2 h at RT. 5 μL of each sample was pooled, desalted, and analyzed by MS to check labeling efficiency. After the labeling efficiency check, samples were quenched by adding 5% hydroxylamine. The pooled samples were then desalted with Strata X C18 SPE column (Phenomenex) and dried by vacuum centrifugation. The samples were fractionated into fractions by high pH reverse-phase HPLC using Agilent 300 Extend C18 column (5 μm particles, 4.6 mm ID, 250 mm length). Briefly, peptides were separated with a gradient of 2% to 60% acetonitrile (ThermoFisher Scientific) in 10 mM ammonium bicarbonate (Sigma-Aldrich) pH 10 over 80 min into 80 fractions. Then, the peptides were combined into 9 fractions and dried by vacuum centrifugation. For enriching modified peptides, tryptic peptides dissolved in NETN buffer (100 mM NaCl, 1 mM EDTA, 50 mM Tris-HCl, 0.5% NP-40, pH 8.0) were incubated with pre-washed pan phosphorylation antibody-conjugated agarose beads (PTM Bio) at 4°C overnight with gentle shaking. Then the beads were washed four times with NETN buffer and twice with H_2_O. The bound peptides were eluted from the beads with 0.1% trifluoroacetic acid (Sigma-Aldrich). Finally, the eluted fractions were combined and vacuum-dried.

For LC-MS/MS analysis, the resulting peptides were desalted with C18 ZipTips (Millipore) according to the manufacturer’s instructions. The peptides were dissolved in solvent A (0.1% formic acid, 2% acetonitrile/in water) and directly loaded onto a reversed-phase analytical column (25 cm length, 75 μm ID). Peptides were separated with a gradient from 5% to 25% solvent B (0.1% formic acid in 90% acetonitrile) over 60 min, 25% to 35% in 22 min, and climbing to 80% in 4 min, then holding at 80% for the last 4 min, all at a constant flowrate of 450 nL/min on an EASY-nLC 1200 UPLC system (ThermoFisher Scientific). The separated peptides were analyzed in Q ExactiveTM HF-X (ThermoFisher Scientific) with a nano-electrospray ion source. The electrospray voltage applied was 2.0 kV. The full MS scan resolution was set to 60,000 for a scan range of 350–1600 *m/z*. Up to 20 of the most abundant precursors were then selected for further MS/MS analyses with 30 s dynamic exclusion. The HCD fragmentation was performed at a normalized collision energy (NCE) of 28%. The fragments were detected in the Orbitrap at a resolution of 30,000. The fixed first mass was set as 100 m/z. The automatic gain control (AGC) target was set at 1E5, with an intensity threshold of 3.3E4 and a maximum injection time of 50 ms.

The resulting MS/MS data were processed using Proteome Discoverer (v2.4.1.15). Tandem mass spectra were searched against the UniProt Mus_musculus_10090_SP_20210721.fasta (17089 sequences) mouse database concatenated with reverse decoy database. Trypsin/P was specified as a cleavage enzyme, allowing up to 2 missing cleavages. The mass tolerance for precursor ions was set as 10 ppm in the first search and 5 ppm in the main search, and the mass tolerance for fragment ions was set as 0.02 Da. Carbamidomethyl on Cys was specified as a fixed modification, and acetylation on the protein N-terminal and oxidation on methionine were specified as variable modifications. FDR was adjusted to < 1%.

For proteomic analysis, different isoform was considered as different proteins for data analysis. For phosphoproteomic analysis, phosphopeptide was used for further analysis, including unique and composite (containing ≥2 phosphorylation sites) forms. The normalized quantification data of all quantified proteins, peptides, or phosphopeptides were consolidated (sum of values) to generate a unique subject ID. The consolidated abundance values were then scaled for each protein or phosphopeptide so that the average abundance was one. Differentially modified peptides were determined by fold change (≥1.2 or ≤ 0.83) and *P*-value (≤ 0.05). Differentially modified proteins contained at least one differentially modified peptide.

### 2.12 Bioinformatics analysis

Subcellular localization annotation of differentially modified proteins was performed using WolF Psort (https://wolfpsort.hgc.jp/).

Gene Ontology (GO) annotation proteome was derived from the UniProt-GOA database (http://www.ebi.ac.uk/GOA/). Proteins were classified by GO annotation based on three categories: biological process, cellular component, and molecular function. For each category, a two-tailed Fisher’s exact test was employed to test the enrichment of the differentially expressed protein against all identified proteins. The GO term with a corrected *P* value < 0.05 was considered significant. Go terms of interest were sorted (Fold change >1.5) and visualized in a bubble diagram.

Protein domain annotation was performed for the identified proteins based on the InterProScan database (https://www.ebi.ac.uk/interpro/).

Kinase prediction was performed using iGPS (https://gps.biocuckoo.cn). Kinase activity was evaluated using the Gene Set Enrichment Analysis (GSEA 4.3.2) method, ranked by normalized enrichment scores (NES) and normalized *P* values. A minimum FDR value of 0.25 was used for GSEA analysis. Protein-kinase interactions were identified and filtered with a minimal confidence score ≥ 0.4 by the SRING database (https://cn.string-db.org/). Kinase-substrate interaction network was visualized using Cytoscape software (https://cytoscape.org/).

Motif analysis was performed using the MOMO tool (https://mitra.stanford.edu/kundaje/marinovg/oak/various/programs/meme_4.12.0/doc/momo.html) based on the Motif-x algorithm (37) with a threshold value of 0.000001. Putative kinases corresponding to motifs were predicted according to the database on the webpage (https://esbl.nhlbi.nih.gov/Databases/Kinase_Logos/).

### 2.13 Immunoprecipitation

Cells were washed twice with cold PBS and lysed by RIPA lysis buffer supplemented with protease and phosphatase inhibitor cocktail (Beyotime). After incubation on ice for 30 min, debris was removed by centrifugation at 12,000 *g* for 10 min. The lysates were immunoprecipitated with anti-lamin A/C (Abclonal) antibody (2.5 μg/ml) for 3–4 h at 4 °C. The immunocomplexes were collected by adding 20 μL of protein A+G agarose beads (Beyotime) and softly rotating at 4°C overnight. Beads were washed 5 times with cold Tris-buffered saline (TBS) (20 mM Tris, 150 mM NaCl). Beads were resuspended in 1 × SDS-PAGE loading buffer and boiled for 10 min. Supernatants were collected for subsequent experiments.

### 2.14 Western blotting

Cell lysates and IP samples were analyzed on 8% or 12% SDS-PAGE gels and transferred onto 0.45 μm PVDF membranes (Millipore). Membranes were blocked in a fast-blocking buffer (HYCEZMBIO) for 10 min at RT. Then, membranes were incubated with the following primary antibodies against lamin A/C (Abclonal), PKCζ (Proteintech), pan phosphoserine/threonine (ECMbio), histone 3 (Abclonal), beta-actin (Servicebio) at a dilution of 1:1,000-1:2,000 in TBST at 4°C overnight, followed by incubation with horseradish peroxidase (HRP)-conjugated secondary antibody for 1 h at RT. HRP signal was developed using SuperPico ECL Chemiluminescence Kit (Vazyme), and western blotting images were captured in the Chemiluminescence Imaging system (Tannon 5200).

### 2.15 Lamin A/C overexpression

Full-length lamin A/C CDS (accession number in NCBI: NM_001002011.3) was amplified and cloned into the pLV3 vector (MiaoLingBio, China) using primer pairs (forward primer 5’- gctagcgaattcgaaggatccATGGAGACCCCGTCACAGC -3’; reverse primer 5’- CTACCCAGCGGCCGCggatccttacatgatgctgcagttctggg-3’). Single-site mutations were generated using primer pairs (S423A: forward primer 5’-AAGCTGGAG GCT TCCGAGAGCCGGAGCAGCTT-3’ and reverse primer 5’-TCGGAAGCCTCCAGCTTGCGCTTTTTGGTGAC -3’; S423D: forward primer 5’-AAGCTGGAG GAT TCCGAGAGCCGGAGCAGCTT-3’ and reverse primer 5’- TCGGAATCCTCCAGCTTGCGCTTTTTGGTGAC-3’) and ClonExpress MultiS One Step Cloning Kit (Vazyme) according to the manufacturer’s instructions. The procedure for lentiviral packaging was referred to the protocol posted online (https://www.addgene.org/protocols/lentivirus-production/). Briefly, HEK 293T cells were transfected with DNA/transfection reagent complex containing 1.64 pmol pLV3, 0.72 pmol pMD2.G, 1.3 pmol psPAX2 and 13 μL PEI Transfection Reagent (MedChemExpress). Lentivirus was harvested at 48 h and 72 h post-transfection by filtering cell supernatants using 0.45 μm polyethersulfone (PES) membrane, followed by virus concentration using Universal Virus Precipitation Kit (Beyotime). RAW264.7 cells were repeatedly infected with lentivirus at 100 MOI with 8 μg/mL polybrene (Beyotime) for 18 h, and polyclonal populations were generated by 3-6 μg/mL puromycin (Beyotime) selection. The western blotting test and fluorescence microscopy verified the overexpression.

### 2.16 Statistical analysis

Statistical analysis was conducted using Prism 8.0 software. Normality and lognormality of column data were tested by Shapiro-Wilk test. For normally distributed data, comparisons between two groups were conducted with two-tailed unpaired t-test, comparisons among three or more groups were performed using ANOVA. *Post hoc* test was conducted according to the test of homogeneity of variance. Data were presented as mean ± standard error of the mean (SEM). *P* values smaller than 0.05 were considered as statistically significant. *, **, *** for *P* values < 0.05, < 0.01, < 0.001, respectively.

## 3 Results

### 3.1 Infective larvae of *Strongyloides stercoralis* trigger DNA release in murine macrophages

**Figure 1:**
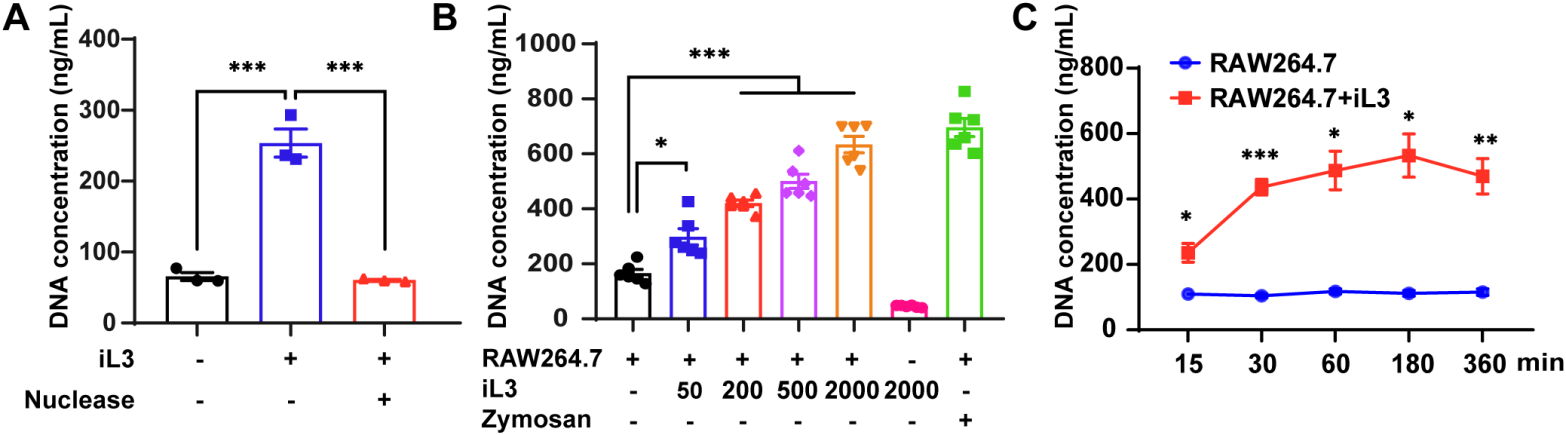
*Strongyloides stercoralis* infective larvae induce DNA release in murine macrophages. **(A)** DNA concentration of supernatants from PMs stimulated with or without S. stercoralis iL3 for 3 h, were quantified using the picogreen dsDNA quantitation kit with a fluorescent microplate reader. The addition of nuclease degraded iL3-induced DNA release. **(B)** iL3 induced DNA release from RAW264.7 macrophage cell line in a dose-dependent manner. RAW264.7 macrophages were exposed to 50, 200, 500, and 2000 iL3 and supernatants were collected for DNA concentration measurement. Supernatants from the cell alone or 2000 iL3 alone were also collected for DNA concentration measurement. Zymosan (250 μg/mL) was set as a positive stimulus of MET induction. **(C)** Comparison of supernatant DNA concentrations between iL3-treated and untreated RAW264.7 macrophages at 15, 30, 60, 180, and 360-minute time points. Data are presented as mean ± SEM (n=3 biological replicates) generated from independent experiments. Statistical significance between groups was assessed by ordinary one-way ANOVA with Tukey’s multiple comparisons test **(A)**, Brown-Forsythe and Welch’s ANOVA with Dunnett’s T3 multiple comparisons test **(B)** and unpaired t-test **(C)**. *P < 0.05, **P < 0.01, ***P < 0.001 between groups are indicated.

Given the robust infiltration of murine macrophages into migratory iL3 microenvironments *in vivo* (38), we established an *in vitro* co-culture system utilizing non-thioglycollate-elicited peritoneal macrophages (PMs) (39, 40) stimulated with sterile iL3 to model early macrophage-nematode interactions. Exposure of PMs to sterile iL3 in the serum-free medium resulted in the formation of fibrous DNA meshworks (**Supplementary Figure 1**), absent in unstimulated cells. Quantification of cell-free double-stranded DNA (dsDNA) in supernatants and nuclease-sensitive degradation confirmed iL3-triggered DNA release (**Figure 1A**).

The high heterogeneity and limited availability of PMs substantially hampered the systematic investigation of MET formation dynamics and underlying mechanisms. Therefore, we deployed RAW264.7, an immortalized macrophage cell line, as a reproducible and tractable model for MET induction. *S. stercoralis* iL3 triggered DNA extrusion from RAW264.7 cells in a dose-dependent manner (**Figure 1B**), and the amount of discharged DNA induced by 2,000 larvae/well was equivalent to zymosan, a known MET inducer (41). Time-course analysis revealed rapid DNA ejection, with over 80% of maximal extracellular DNA release achieved within 30 min (mean=436.5 ng/mL) and peak accumulation occurring within 3 hours (mean=532.9 ng/mL) post-stimulation (**Figure 1C**).

Our data reveal that infective larvae of *S. stercoralis* induce rapid DNA expulsion in murine macrophages.

### 3.2 *Strongyloides stercoralis* iL3-induced extracellular DNA exhibits typical structure and composition of ETs

**Figure 2:**
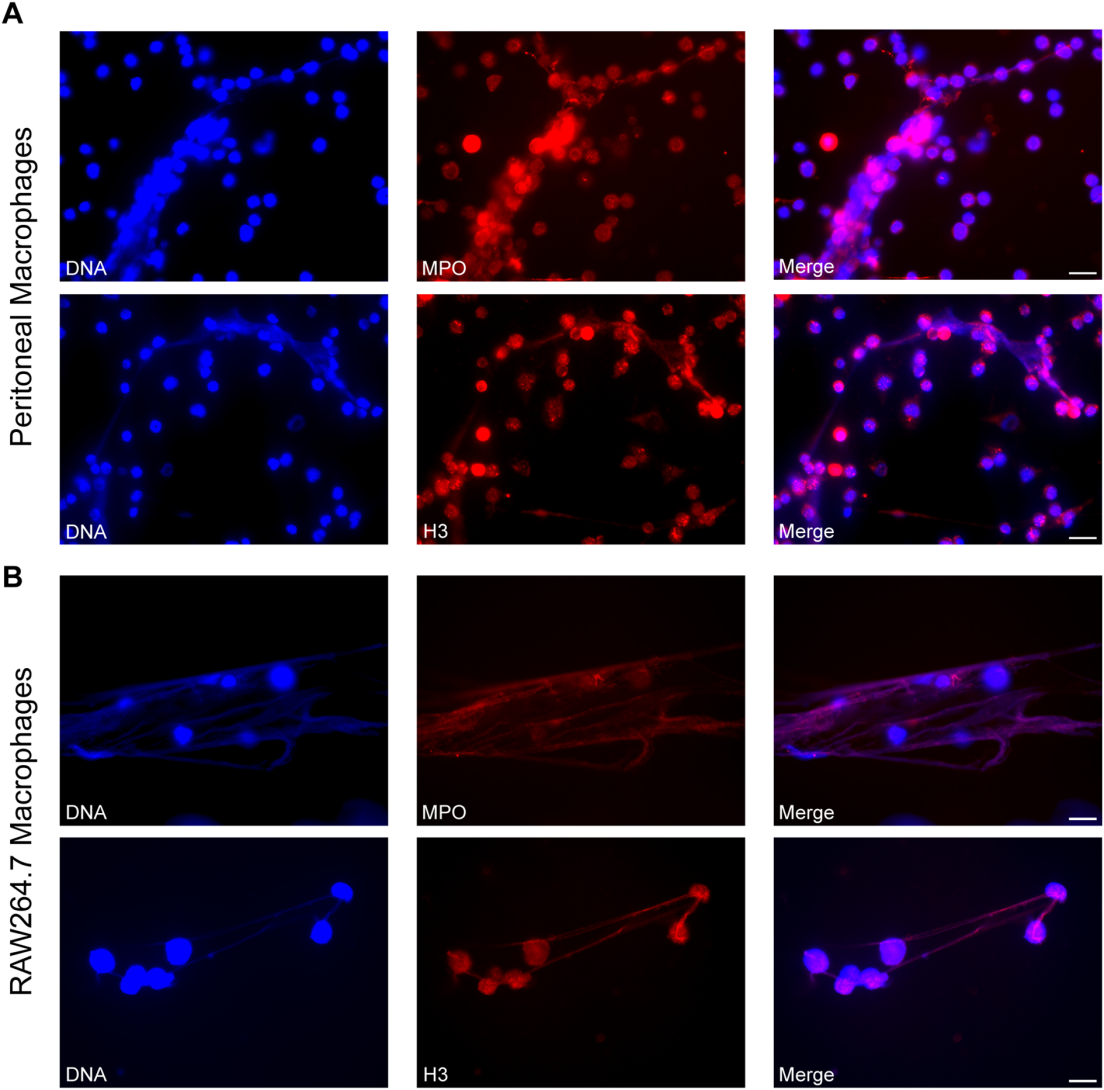
Visualization of METs induced by *Strongyloides stercoralis* iL3. Representative fluorescence microscopy images of METs released by PMs **(A)** and RAW264.7 macrophages **(B)** upon iL3 stimulation for 3 **(H)** DNA was visualized with Hoechst 33258 staining (blue), MPO and H3 were stained with anti-MPO and anti-H3 primary antibodies, respectively, followed by Alexa Fluor 594-labeled secondary antibody (red). Scale bar= 20 μm.

To investigate whether the extracellular DNA induced by *S. stercoralis* exhibits the canonical structural features of ETs, immunofluorescence imaging was performed. Both peritoneal and RAW264.7 macrophages produced fibrous DNA meshwork upon iL3 stimulation for 3h (**Figures 2A, B**). MET identity was confirmed by co-staining of cytoplasmic myeloperoxidase MPO and nuclear histone 3 (H3) (**Figures 2A, B**), hallmarks of canonical extracellular traps (42).

Overall, these findings confirmed that *S. stercoralis* iL3 trigger MET formation in murine macrophages.

### 3.3 *Strongyloides stercoralis*-induced METs originate from nuclear DNA through non-lytic mechanisms

**Figure 3:**
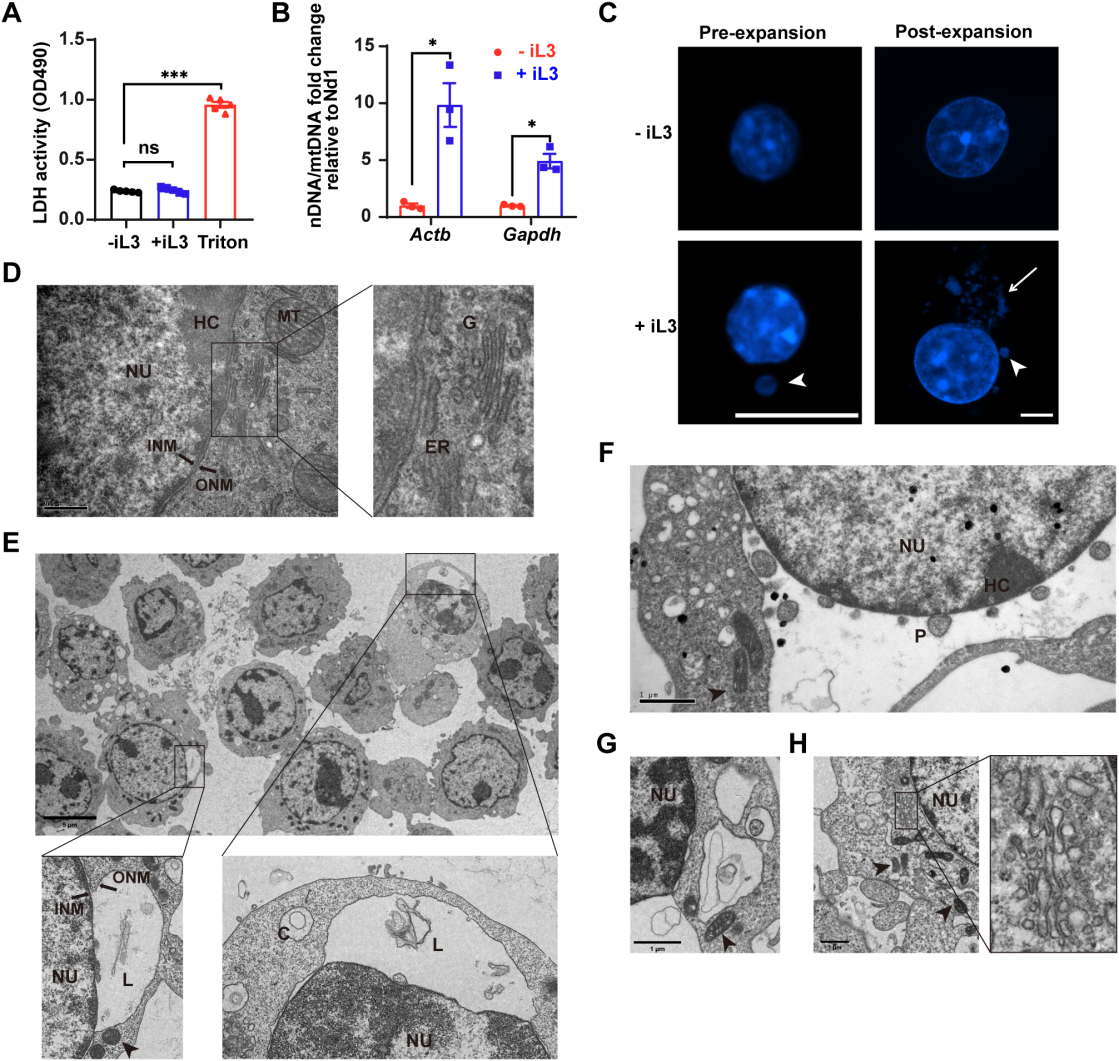
METs are derived from the nucleus with endoplasmic reticulum vacuolation upon *Strongyloides stercoralis* iL3 stimulation. **(A)** Lactate dehydrogenase (LDH) release quantification in supernatants of RAW264.7 macrophages exposed to iL3 (+ iL3) or not (- iL3) for 3 **(H)** Supernatants were collected, and the LDH activities were detected using an LDH Release Assay Kit, followed by absorbance measurement at 490 nm using a microplate reader. Triton X-100 was used as a positive control to lyse cells (Triton). **(B)** qPCR analysis of nDNA/mtDNA ratios in supernatants from iL3-stimulated versus unstimulated RAW264.7 macrophages. Fold changes in nDNA/mtDNA ratios (iL3-stimulated vs. unstimulated) are shown. Nuclear genes (*Actb*, *Gapdh*) were normalized to mitochondrial gene *Nd1*. **(C)** Fluorescence images of intracellular DNA with Hoechst staining. Cells were stimulated with iL3(+iL3)or without iL3 (-iL3) for 15 min, fixed, and stained with Hoechst 33258 (pre-expansion). Before DNA staining, fixed cells were either expanded following the Ultrastructure Expansion Microscopy (U-ExM) procedure (See materials and methods) (post-expansion). Scale bar=10 μm. **(D)** Representative transmission electron microscopy (TEM) image of RAW264.7 without iL3 stimulation. The right panel displays a high-magnification view of the characteristic morphology of the endoplasmic reticulum and Golgi apparatus from the left panel. Scar bar= 0.5 μm. **(E-H)** Representative TEM images of RAW264.7 with iL3 stimulation for 5 min **(F)**, 15 min **(E, G)**, and 30 min **(H)**. Scale bar: **(E)** 5 μm; **(F-H)** 1 μm. C=circular DNA; ER=endoplasmic reticulum; G=Golgi apparatus; HC=heterochromatin; INM=inner nuclear membrane; L=linear DNA; MT=mitochondrion (arrowhead); NU=nuclei; ONM=outer nuclear membrane; P=DNA-containing particle. Data are presented as mean ± SEM (n=5 biological replicates for panel **A**; n=3 for panel **B**), generated from independent experiments. Statistical significance was assessed by Brown-Forsythe and Welch’s ANOVA with Tamhane’s T2 multiple comparisons test for **(A)**. Unpaired t-test with Welch’s correction was performed to compare the adjacent columns **(B)**. ns, not significant, *P < 0.05, ***P < 0.001 between groups are indicated.

*Strongyloides*-induced MET formation by murine macrophages and RAW264.7 cells provided a model to investigate the cellular mechanism. The nuclear envelope (NE) disassembly and plasma membrane permeabilization are hallmarks of lytic nuclear DNA release during classical NET formation (42). In contrast, *S. stercoralis* iL3 stimulation for 3 h did not elevate lactate dehydrogenase (LDH) activity in cell supernatants (**Figure 3A**), indicating preserved plasma membrane integrity during MET formation. This result was corroborated by propidium iodide (PI) exclusion assays (**Supplementary Figure 2A**), confirming the absence of significant plasma membrane permeability changes.

Prior studies have established that mitochondrial DNA can be rapidly released to form ETs in neutrophils (43) and eosinophils (44) without cell lysis. To investigate whether similar mechanisms underlie MET formation, we analyzed the origin of *S. stercoralis* iL3-induced METs. Although both mitochondrial (*Atp6, Nd1*) and nuclear (*Actb, Gapdh*) genes were detectable in cell supernatants (**Supplementary Figure 3**), quantitative real-time PCR (qPCR) demonstrated significant enrichment of nuclear DNA markers over mitochondrial counterparts (*Nd1*: **Figure 3B**; *Atp6*: **Supplementary Figure 2B**) following iL3 stimulation for 3 h, establishing nuclear DNA as the primary source of METs.

Ultrastructure analysis further confirmed the nuclear origin of METs. Conventional immunofluorescence assay with DNA staining detected a DNA particle localized within the iL3-stimulated cell (**Figure 3C**, pre-expansion), while expansion microscopy (3-4×physical expansion) resolved abundant perinuclear DNA aggregates (**Figure 3C**, post-expansion). Strikingly, transmission electron microscopy (TEM) imaging revealed a large separation between the inner and outer nuclear membrane (INM/ONM) upon iL3 stimulation for 5 min with DNA fragments or vesicles in the dilated perinuclear space (**Figures 3E, F**). These critical morphological features distinguished this process from mitotic NE breakdown (**Supplementary Figure 2C**). In addition, iL3-stimulated cells displayed disintegrated and vacuolated endoplasmic reticulum (ER) in the cytoplasm with iL3 stimulation (**Figures 3E–G**, **Supplementary Figure 2E**), unlike the well-organized tubular structures in cells without larval exposure (**Supplementary Figure 3D**). DNA fragments and particles were also present in vacuolated ER (**Figure 3G**). Noteworthily, within 30 min, the vacuolar ER underwent a reorganization into a tubular structure (**Figure 3H**), concomitant with the restoration of INM/ONM separation (**Figure 3H**).

The TEM imaging also confirmed the overall integrity of the NE and plasma membrane (**Figures 3E–H**). Moreover, the distinctive heterochromatin underlying the INM indicated the maintenance of heterochromatin architecture, excluding global decondensation (**Figures 3E–H**). Concurrently, the mitochondria displayed remarkable ultrastructural changes, including cristae loss and increased electron density (**Supplementary Figure 2E**), as well as a transition to elongated or compact morphologies (**Figures 3F, H**).

In brief, *S. stercoralis*-induced METs are formed rapidly with distinctive ultrastructural alterations in the NE, ER, and mitochondria, which lead to a non-lytic discharge of the nuclear DNA release process.

These coordinated nuclear and cytoplasmic alterations demonstrate that *S. stercoralis* induces rapid, non-lytic MET formation through NE remodeling rather than classical lytic pathways.

### 3.4 *Strongyloides*-induced MET formation does not require NADPH oxidase, reactive oxygen species, MPO, neutrophil elastase (ELNE), or Ca²^+^

The distinct ultrastructural features of *S. stercoralis*-induced MET formation prompted systematic investigation of their molecular regulation. Considering that NADPH oxidase, ROS, MPO, elastase, and Ca²^+^ are essential to produce NETs, we tested their requirement in *S. stercoralis*-induced MET formation through pharmacological inhibition (45). Firstly, diphenyleneiodonium chloride (DPI) failed to suppress DNA release (**Supplementary Figure 4A**), indicating that the parasite-induced MET formation is NOX-independent. Furthermore, the dependency on NOX varied depending on different stimuli, including lipopolysaccharide (LPS) (a component of the outer wall from gram-negative bacteria), *Mycobacterium smegmatis* MC^2^155 strain (a gram-positive bacterium) and zymosan (an insoluble β-glucan-rich particle of cell wall from *Saccharomyces cerevisiae*) (**Supplementary Figure 4B**). Since NOX is not the only source of intracellular ROS (46), a potent antioxidant N-acetylcysteine (NAC) was used to scavenge global ROS, which likewise failed to attenuate MET formation (**Supplementary Figure 4C**). Critically, even when blocking the downstream effectors of the NOX-ROS axis—MPO and ELNE, MET production remained unaffected (**Supplementary Figure 4D**), providing additional evidence that MET generation occurs independently of this pathway. In addition, neither chelation of extracellular Ca²^+^ (via EGTA) nor intracellular Ca²^+^ (via BAPTA-AM) reduced MET release, indicating that Ca²^+^ signaling is dispensable for MET formation (**Supplementary Figure 4E**). These collective findings demonstrate that murine macrophages release METs in response to *S. stercoralis*, employing a distinct mechanism independent of NOX-ROS-MPO/ELNE cascade or Ca²^+^ flux.

### 3.5 *Strongyloides* iL3-exposed macrophages exhibit only a modest change in protein levels

Ultrastructural analysis revealed early subcellular changes, including nuclear membrane separation and ER fragmentation within 5 min of iL3 stimulation (**Figures 3E–G**), preceding detectable extracellular DNA release at 15 min (**Figures 2B**, **3C**). This compressed timeline suggested that *S. stercoralis-*induced MET formation is independent of *de novo* gene expression. To investigate whether transcription is required for MET formation, RAW264.7 macrophages were treated with RNA polymerase II inhibitors actinomycin D before iL3 stimulation. We first confirmed the activity of actinomycin D and determined the concentrations required for transcriptional inhibition. Zymosan is known as an inducer of tumor necrosis factor (TNF-α) *de novo* production (47, 48). 1 μg/mL of actinomycin D potently inhibited zymosan-elicited *Tnf-α* gene transcription (**Supplementary Figure 5A**), while it was unable to significantly suppress MET production (**Supplementary Figure 5C**). However, a high concentration of actinomycin D (5 μg/mL) partially attenuated MET release without affecting the cell viability (**Supplementary Figures 5B, C**).

To systematically profile cellular protein alteration, quantitative proteomic analysis was conducted comparing iL3-stimulated and unstimulated RAW264.7 macrophages. Only 54 differentially regulated proteins (FC≥1.2-fold), 24 up-regulated and 30 down-regulated, were identified (see the top 10 up- and down-regulated proteins listed in **Table 1**). Among them, properdin (P11680, 0.69), interferon-induced transmembrane protein 3 (Q9CQW9, 0.708), CD82 antigen (P40237, 0.742), and DDB1- and CUL4-associated factor 15 (Q6PFH3, 1.613) are involved in immune response. UBX domain-containing protein 8 (Q9QZ49, 0.712), ER lumen protein-retaining receptor 3 (KDELR3) (Q8R1L4, 1.368), and gamma-aminobutyric acid receptor-associated protein-like 2 (P60521, 0.734) are ER or Golgi proteins that may be involved in autophagy (49–51). The extracellular matrix (ECM) protein fibronectin (Fn1) (P11276, 0.46) binds the macrophage surface participating in cell adhesion, maintenance of cell shape, macrophage polarization, and activation (52). Nuclear proteins homologous recombination OB-fold protein (HROB) (Q32P12, 0.766) and Zinc finger protein 219 (Q6IQX8, 1.34) regulate DNA repair and transcription, respectively. Overall, the protein landscape of iL3-exposed macrophages was only negligibly perturbed.

**Table 1:** Top 10 up- and down-regulated proteins in RAW264.7 cells exposed to iL3 of *Strongyloides stercoralis* compared to unstimulated cells.

Entry	Protein name	Fold change	*P* value
P11276	Fibronectin	0.46	0.000153
P11680	Properdin	0.69	0.0008177
Q9CQW9	Interferon-induced transmembrane protein 3	0.708	0.0002539
Q9QZ49	UBX domain-containing protein 8	0.712	0.0373346
Q9CR83	Probable RNA-binding protein 18	0.717	0.0106931
Q9Z222	N-acetyllactosaminide beta-1,3-N acetylglucosaminyltransferase 2	0.718	0.0288965
Q8VDY4	EF-hand calcium-binding domain-containing protein 7	0.721	0.0087435
P60521	Gamma-aminobutyric acid receptor-associated protein-like 2	0.734	0.0484029
P40237	CD82 antigen	0.742	0.0133511
Q32P12	Homologous recombination OB-fold protein	0.766	0.0282762
Q61193	Ral guanine nucleotide dissociation stimulator-like 2	1.266	0.0238548
Q8BGC1	UPF0489 protein C5orf22 homolog	1.273	0.018626
O88851	Putative hydrolase RBBP9	1.279	2.142E-05
P62858	40S ribosomal protein S28	1.294	0.0016468
Q6IQX8	Zinc finger protein 219	1.34	0.0157458
Q9D011	M-phase-specific PLK1-interacting protein	1.353	0.0277659
P02802	Metallothionein-1	1.366	0.006821
Q8R1L4	ER lumen protein-retaining receptor 3	1.368	0.0012996
Q8K039	Uncharacterized protein KIAA1143 homolog	1.467	0.0001942
Q6PFH3	DDB1- and CUL4-associated factor 15	1.613	0.0077772

Entry: protein entry in Uniprot database (https://www.uniprot.org/)

### 3.6 Phosphoproteomics reveals molecular machineries in *S. stercoralis*-stimulated macrophages

Given the limited proteomic changes and rapid MET kinetics, our extended work studied protein phosphorylation by tandem mass tag (TMT)-based comparative phosphoproteomics of iL3-stimulated and unstimulated RAW264.7 cells (**Supplementary Figure 6**). A total of 9709 phosphorylated peptides corresponding to over 3521 proteins were detected, of which 538 proteins with 927 sites were down-regulated and 320 proteins with 488 sites were up-regulated (**Supplementary Figures 6A, D**).

**Figure 4:**
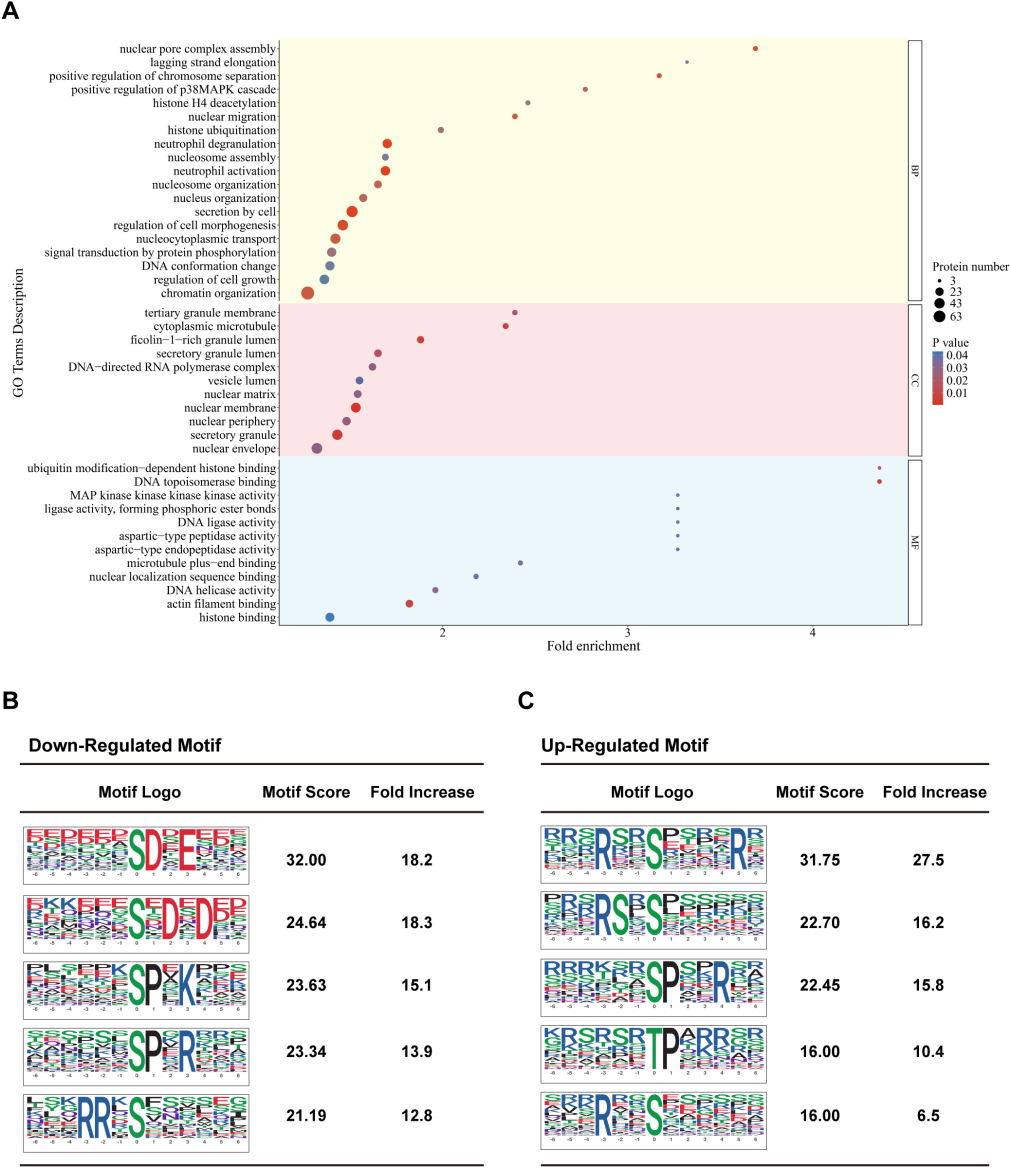
Comparative phosphoproteomics analysis of *Strongyloides stercoralis* iL3-stimulated macrophages. **(A)** Gene ontology (GO) enrichment analysis of differentially modified proteins comparing iL3-stimulated versus non-stimulated RAW264.7 macrophages. Shown are selected significantly enriched GO terms (*P* ≤ 0.05) distributed in three categories: biological process (BP), cellular component (CC), and molecular function (MF). **(B, C)** Top 5 enriched phosphorylation motifs for **(B)** down-regulated and **(C)** up-regulated phosphosites. Motif logos represent amino acid preferences (± 6 residues) around phosphorylated Ser/Thr/Tyr (S/T/Y) sites. Motifs are ranked by both motif score (indicating statistical significance and specificity) and fold enrichment.

Bioinformatic analysis revealed nuclear-centric regulation. First, 65.81% of differentially modified proteins (DMPs) were localized in the nucleus (**Supplementary Figure 6F**). Next, DMPs were categorized into biological process, cellular component, and molecular function (**Figure 4A**, **Supplementary Figure 7A–C**) by Gene Ontology (GO) annotation. We observed enrichment in nuclear compartments, including NE, nuclear periphery, nuclear membrane, nuclear matrix, nuclear pore complex assembly (**Figure 4A**), nuclear speck, and nuclear pore nuclear basket (**Supplementary Figure 7A**). Enrichment of the GO terms, such as chromatin organization, DNA conformation change, nucleocytoplasmic transport, nucleus organization, histone deacetylation and ubiquitylation implied DMPs’ role in nuclear structural and functional regulation (**Figure 4A**). Unexpectedly, GO terms associated with transcription (DNA-directed RNA polymerase complex, RNA processing, RNA splicing, RNA polymerase core enzyme binding, etc.) were predominately enriched (**Figure 4A**; **Supplementary Figure 7B**). Likewise, protein domain enrichment analysis highlighted a strong association with RNA recognition and metabolism (**Supplementary Figure 7D**). In addition, the enrichment of bromodomain-containing proteins, which recognize histone acetylation and regulate transcription (53), revealed potential roles of histone post-translational modification and chromatin remodeling (**Supplementary Figure 7D**).

Furthermore, the enrichment analysis revealed the regulation of MAPK (mitogen-activated protein kinase) and AKT (Protein kinase B) cascade (**Figure 4A**, **Supplementary Figure 7C**) in *S. stercoralis* iL3-stimulated macrophages. The occurrence of other terms, cytoplasmic microtubule, microtubule plus-end binding, actin filament binding (**Figure 4A**), cortical microtubule, kinetochore microtubule (**Supplementary Figure 7A**), profilin binding (**Supplementary Figure 7C**), indicated that exposure to *S. stercoralis* iL3 led to the arrangement of microfilament and microtubule cytoskeleton in macrophages.

Motif analysis was performed to illustrate the preference for amino acid residues flanking the identified phosphorylated serine/threonine sites (S/T) and to obtain added insight into differentially modified peptides. The significantly enriched motifs of down-phosphorylated peptides included aspartic acid (D)-directed phosphorylation, in which [XXX-(S)DXEX] corresponds to the substrate motif of casein kinase CK2, and [XXX(S)XDXD] corresponds to the substrate motif of Ca^2+^/calmodulin-dependent protein kinase 2 delta/gamma (CAMK2D/G). The motif [XXX(S/T)PX-K/R-XX] is a characteristic motif of cyclin-dependent kinases (CDKs) targeting sequences (**Figure 4B**). The significantly enriched motifs of up-phosphorylated peptides correspond to arginine (R) directed phosphorylation [RXX(S/T)XXX] (**Figure 4C**). Additionally, proline (P)-directed phosphorylation motifs containing positively-charged amino acids (lysine/arginine, K/R) at the +3 site were enriched in down-phosphorylated sequences, while those at the +4 site were enriched in up-phosphorylated sequences. (**Figure 4B, C**).

### 3.7 The AKT and ERK signaling networks regulate the MET formation

**Figure 5:**
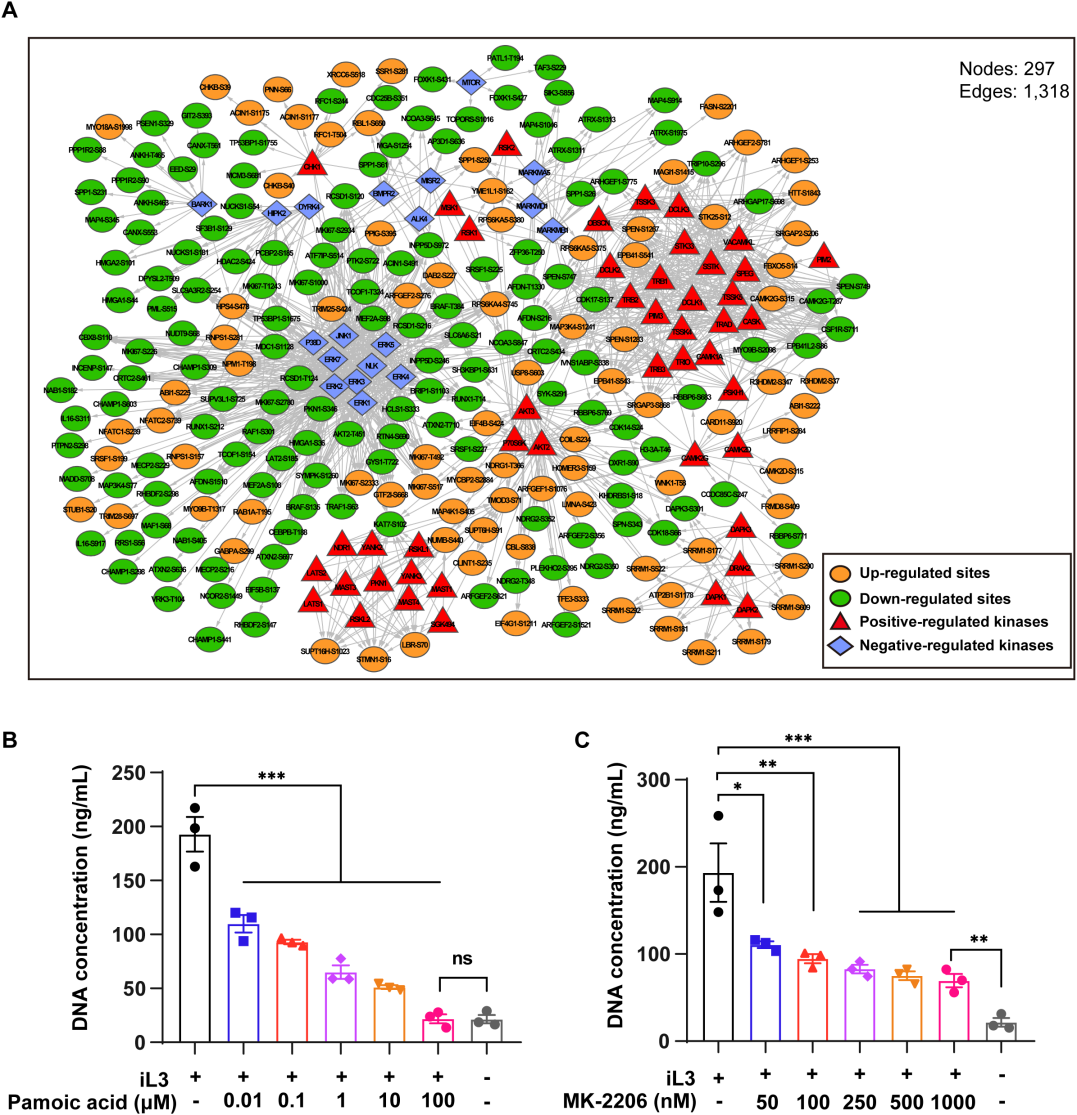
The interaction network of identified substrates and predicted kinases revealed the central role of ERK and AKT. **(A)** Interaction network of identified differentially modified sequence substrates and corresponding predicted kinases. Substrates were sorted according to GO annotation and enrichment. Significant subsets (*P* < 0.05) were extracted for constructing subnetworks. Shown are subnetworks and different color lumps. **(B, C)** Quantitative analysis of iL3-induced MET release with pretreatment with ERK agonist pamoic acid **(B)** and AKT inhibitor MK-2206 **(C)** at indicated concentrations. DMSO was used as a vehicle control and solvent for the inhibitors. Data are presented as mean ± SEM of 3 biological replicates, generated from independent experiments. Statistical significance was analyzed by one-way ANOVA with Tukey’s multiple comparisons test. ns, not significant, *P < 0.05, **P < 0.01, ***P < 0.001.

To gain insight into kinase-substrate interaction, kinases were predicted using the GPS 6.0 algorithm (54), followed by filtration with the STRING database. First, the kinase activity in iL3-stimulated cells, predicted as positively or negatively regulated, was assessed by GSEA enrichment (**Supplementary Figure 8**). Next, all the predicted kinases and differentially modified sites were used to construct a kinase-substrate interaction network, revealing ERK and AKT as central regulatory hubs with inverse activity patterns: ERK activity decreased while AKT increased during MET formation (**Figure 5A**). Based on GO classification and enrichment, sub-networks were generated targeting the cytoskeleton, endomembrane system, chromatin organization, signaling transduction, and cell death (**Supplementary Figure 9**).

In further work, we searched phosphoproteomics data for known regulators that directly/indirectly regulate upstream members of the ERK (**Table 2**) and AKT cascade (**Table 3**). Dual phosphorylation of ERK1 at T203/Y205, essential for activation of ERK1 (55), was up-regulated in *S. stercoralis*-stimulated macrophages (**Table 2**). Five down-regulated phosphorylation sites were identified in two RAF protein kinases, B-RAF (S135, S431, T384) and C-RAF (also known as RAF1; S301, T638). A previous report suggested that phosphorylation of C-RAF at S301 represents a feedback mechanism dependent on ERK activity, which leads to decreased C-RAF activity (56). Next, RAW264.7 macrophages were pretreated with pamoic acid, a specific ERK agonist (57), before iL3 stimulation to determine ERK’s role in MET formation. Indeed, pamoic acid inhibited *S. stercoralis*-triggered DNA release in a dose-dependent manner (**Figure 5B**).

**Table 2:** Changes in phosphorylation of proteins regulating ERK signaling cascade in RAW264.7 cells exposed to iL3 of *Strongyloides stercoralis* compared to unstimulated cells.

Protein ID	Protein name	Amino acid	Position	IL3/control ratio
Q99N57	RAF1	T	638	0.821
		S	301	0.823
P34152	PTK2	S	722	0.821
Q9WUU8	TNIP1	S	441	0.815
Q6PHZ2	CAMK2D	S	315	1.306
Q923T9	CAMK2G	S	315	1.301
		T	287	0.812
P97492	RGS14	S	458	1.216
Q06180	PTPN2	S	298	0.819
		S	320	1.214
P83741	WNK1	T	58	1.207
P58801	RIPK2	S	364	1.348
P28028	BRAF	T	384	0.78
		S	135	0.807
		S	431	0.778
Q60875	ARHGEF2	S	781	1.557
P09581	CSF1R	S	711	0.811
P15379	CD44	T	726	0.82
Q8BZ03	PRKD2	S	197	0.822
Q4JIM5	ABL2	S	632	0.819
		S	671	0.81
P98078	DAB2	S	227	1.204
Q63844	MAPK3 (ERK1)	T	203	1.243
		Y	205	1.243
P48025	SYK	S	291	0.831
Q9QYG0	NDRG2	S	350	0.74
		T	348	0.74
		S	352	0.729
Q8BHL3	TBC1D10B	T	136	0.785
		S	644	0.822
Q8K3G5	VRK3	T	104	0.783
O08586	PTEN	S	385	0.579

Proteins regulating the RAS-RAF-MEK-ERK signaling cascade were identified and sorted by GO annotation (*P* < 0.05). Reference GO terms: ERK1 and ERK2 cascade (GO:0070371); regulation of ERK1 and ERK2 cascade (GO:0070372); positive regulation of ERK1 and ERK2 cascade (GO:0070374).

**Table 3:** Changes in phosphorylation of proteins regulating AKT signaling cascade in RAW264.7 cells exposed to iL3 of *Strongyloides stercoralis* compared to unstimulated cells.

Protein ID	Protein name	Amino acid	Position	IL3/control ratio
Q60823	Akt2	T	451	0.805
Q3U182	Crtc2	S	461	0.822
		S	70	0.78
		S	434	0.798
P09581	Csf1r	S	711	0.811
Q8BGD9	Eif4b	S	424	1.306
		S	425	1.462
Q9Z1E4	Gys1	T	722	0.773
		S	718	0.773
O35664	Ifnar2	S	444	0.829
Q6RHR9	Magi1	S	1415	1.323
Q63844	Mapk3	T	203	1.243
		Y	205	1.243
P70268	Pkn1	S	346	0.826
Q8BWW9	Pkn2	T	124	1.491
O08586	Pten	S	385	0.579
P34152	Ptk2	S	722	0.821
Q99N57	Raf1	T	638	0.821
		S	301	0.823
P62754	Rps6	S	82	0.797
P10923	Spp1	S	250	1.342
		S	212	0.828
		S	231	0.672
		S	61	0.691
		S	26	0.709
		S	27	0.749
Q61037	Tsc2	S	1343	0.821
Q9ERV1	Mkrn2	S	365	1.358

Proteins regulating the PI3K-AKT signaling cascade were identified and sorted by based on KEGG annotation (*P* < 0.05). Reference KEGG pathway: PI3K-AKT signaling (map04151).

The activity of AKT kinases was predicted to be up-regulated during MET formation (**Figure 5A**). AKT family kinases comprise three closely related members (AKT1, AKT2 and AKT3), whose regulatory activation was achieved by phosphoinositide 3-kinase (PI3K)-derived production of PtdIns-3,4-P2 (PI3,4P2) and PtdIns-3,4,5-P3 (PIP3). Conversely, phosphatase and tensin homolog (PTEN) catalyze a reverse reaction and negatively regulate AKT activity (58). We identified down-regulated phosphorylation at T451 in AKT2 with an unknown function (**Table 3**). It was reported that constitutive phosphorylation at the C-tail region of PTEN, including S385, by casein kinase 2 (CK2), contributes to the stability of PTEN (59, 60). We observed that the decreased phosphorylation of PTEN at S385 was consistent with upregulated AKT activity during MET formation. The involvement of AKT signaling in MET formation was conclusively determined by chemical inhibition using a specific inhibitor MK-2206. Pretreatment with MK-2206 attenuated MET release induced by iL3 (**Figure 5C**).

These findings indicated that MET release is positively controlled by the AKT while negatively regulated by the ERK signaling cascade.

### 3.8 Histone acetylation facilitates MET formation

**Figure 6:**
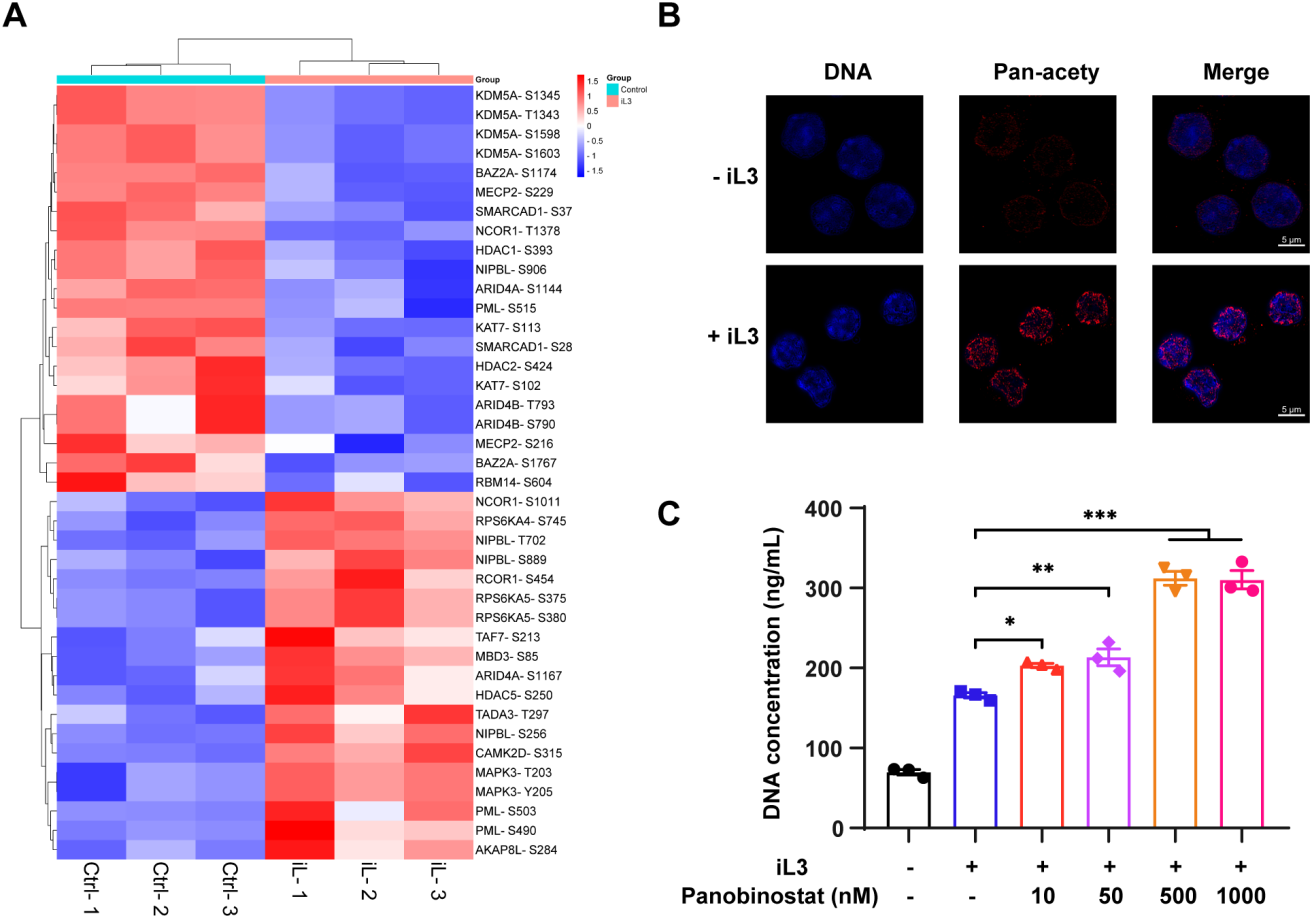
Histone acetylation promotes MET release. **(A)** Heatmap of differentially modified proteins involved in regulating histone acetylation. The heatmap was generated based on GO annotation. Proteins are displayed as the abbreviation of protein names with identified modified sites. **(B)** Representative confocal fluorescence images of RAW264.7 macrophages with (+iL3) or without (-iL3) stimulation for 15 min. Cells were fixed and stained with anti-pan acetylation monoclonal antibody, followed by 594-labeled secondary antibody staining (red). DNA was stained with Hoechst 33258 (blue). Images were captured under the same acquisition model settings. Scar bar = 5 μm. **(C)** Quantitative analysis of iL3-induced MET release in the absence or presence of HDAC inhibitor panobinostat. Cells were pretreated with panobinostat for 30 min at 10, 50, 500, and 1000 nM, followed by iL3 stimulation for 3 **(H)** Cell supernatants were collected for DNA concentration quantification. Data are presented as mean ± SEM of 3 biological replicates, generated from independent experiments. Statistical significance was analyzed by one-way ANOVA with Dunnett’s multiple comparisons test. *P < 0.05, **P < 0.01, ***P < 0.001.

GO analysis indicated that chromatin remodeling and histone deacetylation in MET formation (**Figure 4A**) was supported by enrichment of bromodomains (histone acetylation readers) and histone deacetylase (HDAC) domains among DMPs (**Supplementary Figure 7D**). The kinase-substrate interaction networks further connected histone modification to chromatin reorganization (**Supplementary Figure 9**), indicating the involvement of histone acetylation in MET formation. Heatmap shows key regulators of acetylation dynamics, including histone deacetylase (HDAC1, HDAC2, and HDAC5), histone acetyltransferase (KDM5A and KAT7), and other chromatin remodeling factors that recruit/regulate HDAC, such as BAZ2A, MECP2, NCOR1, PML, and SMARCAD1 (**Figure 6A**). Indeed, histone acetylation significantly increased in the nucleus following stimulation with iL3 (**Figure 6B**) as confirmed by immunofluorescence assay. Furthermore, acetylated histone was robustly enriched in nucleus-derived DNA vesicles (**Figure 6B**). Consistently, pan-HDAC inhibitor panobinostat promoted *S. stercoralis*-induced MET release (**Figure 6C**) while reducing the basal discharge of DNA slightly without affecting cell viability (**Supplementary Figures 10A, B**). In summary, we conclude the role of histone acetylation in regulating MET formation.

### 3.9 MET formation involves remodeling of the F-actin cytoskeleton

DMPs were significantly associated with the molecular function of actin filament binding (**Figure 4A**), with RHO GTPase signaling pathways emerging as central regulators in *S. stercoralis* iL3-stimulated macrophages (**Supplementary Figure 7E**). RHO GTPases (one of the Ras-related superfamily of small GTPases) are known to modulate organization (61, 62).

**Figure 7:**
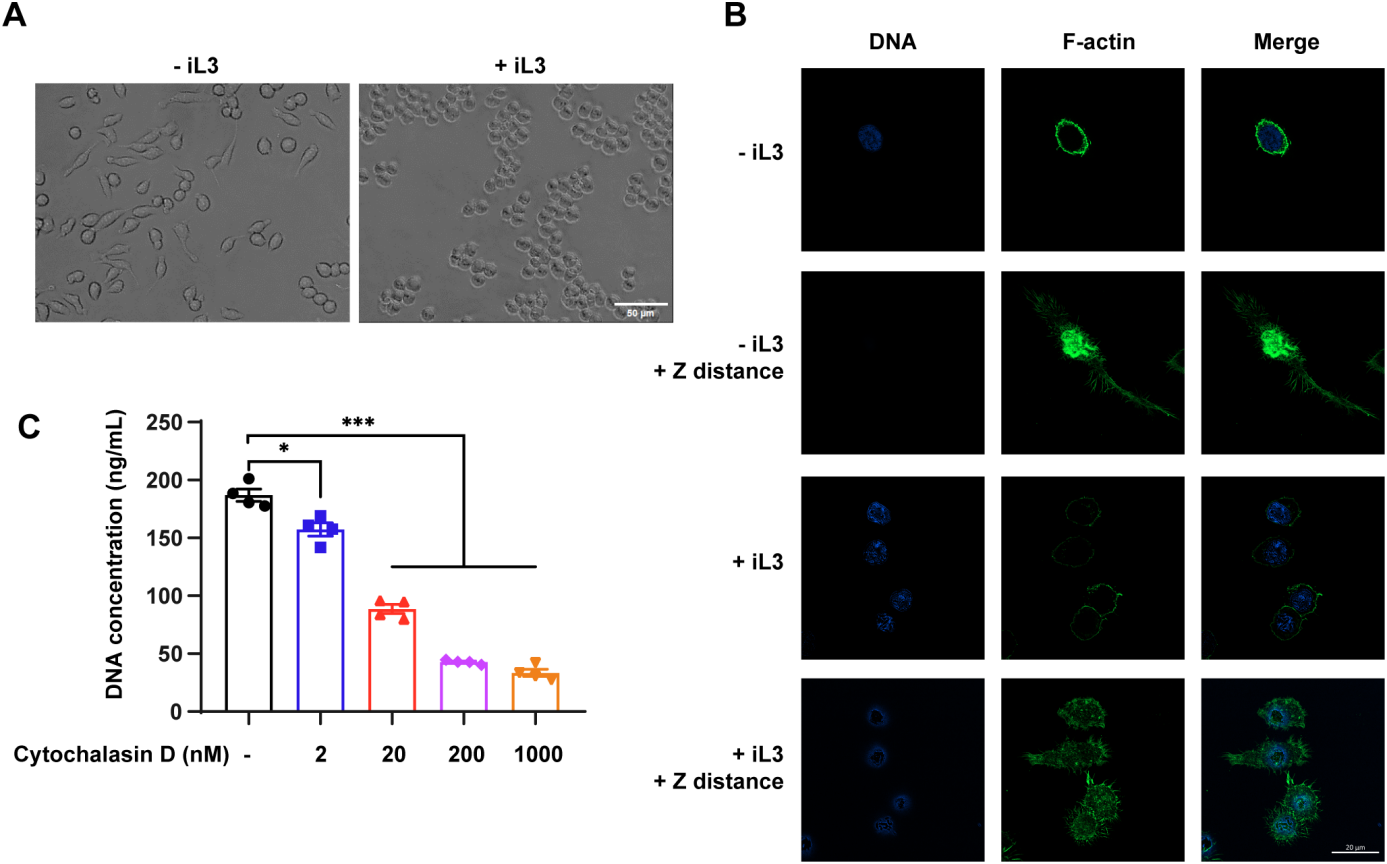
F-actin rearrangement is involved in MET formation. **(A)** Bright-field images of RAW264.7 incubated with iL3 (+iL3) or not (-iL3) in serum-free medium for 3 **(H)** Scale bar=50μM. **(B)** Representative confocal microscopy images of RAW264.7 incubated with iL3 (+iL3) or not (-iL3) in serum-free medium stained with Hoechst for DNA (blue) and AbFluor™ 488-labeled phalloidin for F-actin (Green). For a single cell, one image was captured focused on the nucleus (in the optical plane at the ventral cell surface), and another was captured with increased Z axis distance (≈3 μm), where F-actin is mainly distributed or concentrated (+Z distance). Scale bar=20 μM. **(C)** Quantitative analysis of MET release in RAW264.7 macrophages that were pretreated without or with cytochalasin D at the indicated concentration for 30 min. After pretreatment with Cytochalasin D, cells were exposed to iL3 for 3 h, and supernatants were collected for DNA quantification. Data are presented as mean ± SEM of 4 biological replications generated from independent experiments. Brown-Forsythe and Welch ANOVA with Dunnett’s T3 multiple comparisons test was performed for statistical analysis. *P < 0.05, ***P < 0.001.

The unstimulated macrophages showed a mixture of spindle-shaped and elongated appearances (**Figure 7A**, -iL3) while most cells transformed into rounded morphology upon iL3 stimulation (**Figure 7A**, +iL3). Given that actin plays a central role in maintaining cell shape and polarity (63), we assessed F-actin distribution change in parasite-stimulated macrophages. F-actin exhibited prominent perinuclear localization in control cells (**Figure 7B**, -iL3), which declined upon iL3 exposure (**Figure 7B**, +iL3). In addition, unstimulated macrophages harbored F-actin in the central region, distributed through filopodia and the long axis of the elongated cell body (**Figure 7B**, -iL3, +Z distance). In contrast, iL3 stimulation decreased cell polarity, and F-actin formed clustered podosome-like structures (**Figure 7B**, +iL3, +Z distance). Cytochalasin D was used to inhibit actin polymerization to determine the functional importance of F-actin in MET formation. Indeed, cytochalasin D attenuated MET release in a dose-dependent manner (**Figure 7C**).

Besides, phosphoproteomics also suggested the role of microtubule cytoskeleton organization in MET formation (**Figure 4A**, **Supplementary Figures 7**, **9**). However, pretreatment of RAW264.7 macrophages with taxol did not affect MET release in response to iL3 stimulation (**Supplementary Figure 11**).

### 3.10 PKCζ-mediated lamin A/C phosphorylation drives MET release

The expansion of perinuclear space and the budding of nuclear vesicles indicated a marked regulation of the nuclear envelope (NE). Likewise, several DMPs, including nuclear pore complex (NPC) proteins (NUP50, NUP93, NUP98, PO210, NU214, NDC1, PO121), members of the linker of nucleoskeleton and cytoskeleton (LINC) complex (SYNE1, SUN2), and INM protein (MAN1, EMD) are localized on NE (**Supplementary Figure 12A**). In particular, we noticed an up-regulated phosphorylation in lamina protein lamin A/C (LMNA) at S423. Besides, lamina-associated polypeptide 2 beta (LAP2B) and lamin B receptor (LBR) (**Supplementary Figure 12A**) interact with lamin B and are crucial for heterochromatin localization at the nuclear periphery (64). All these results indicate the structural and functional modulation of NE in macrophages undergoing MET formation.

**Figure 8:**
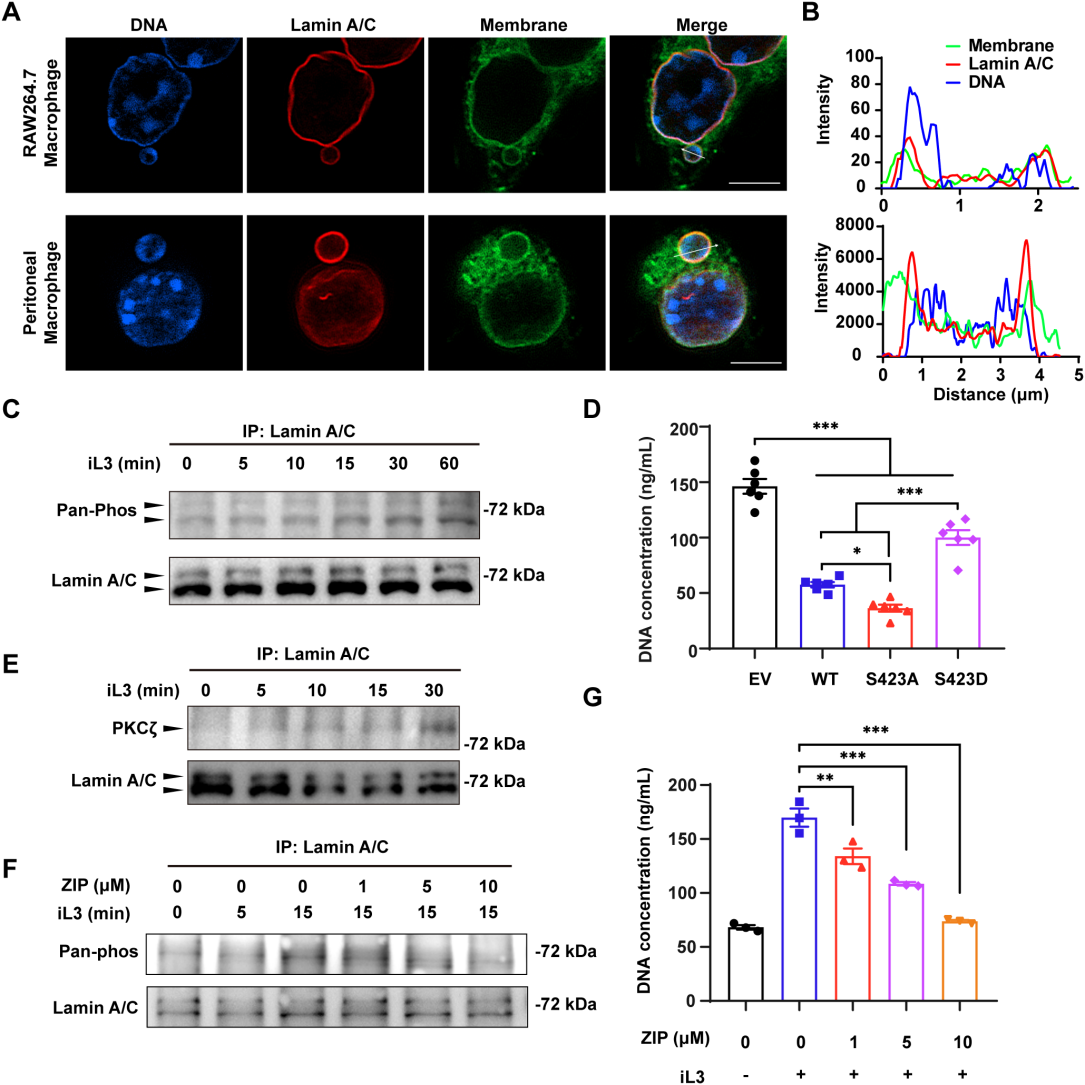
PKCζ-mediated lamin A/C phosphorylation facilitates MET formation. **(A)** Confocal microscopy of RAW264.7 (upper panel) and peritoneal macrophages (lower panel) that were exposed to iL3 for 15 min. Cells were stained with anti-lamin A/C antibody (red), FITC-labeled ConA (green), and Hoechst 33258 (blue). Scale bar=5 μM. **(B)** Fluorescence distribution on the arrow across the vesicles in iL3-stimulated RAW264.7 macrophage (**A**, upper panel) and peritoneal macrophage (**B**, lower panel). **(C)** Representative western blot detection of phosphoserine/threonine and total lamin A/C with lamin A/C protein immunoprecipitated from RAW264.7 cells that were exposed to iL3 for the indicated periods. The PVDF membrane was first probed with an anti-pan phosphoserine/threonine antibody, stripped, and subsequently reprobed with an anti-lamin A antibody to confirm target protein enrichment. **(D)** Quantitative analysis of iL3-induced MET release by RAW264.7 with overexpression of lamin A/C wild-type form (WT), serine-alanine mutation (S423A), or serine-aspartate mutation (S423D) at 423 serine. RAW264.7 transfected with pLV3 empty vector (EV) served as a control. Cells were exposed to iL3 in serum-free medium for 30 min and supernatants were collected for DNA concentration determination. **(E)** Representative western blot detection of PKCζ and total lamin A/C with lamin A/C protein immunoprecipitated from RAW264.7 cells that were exposed to iL3 for the indicated periods. The PVDF membrane was first probed with an anti-PKCζ antibody, stripped, and subsequently reprobed with an anti-lamin A antibody to confirm target protein enrichment. **(F)** Representative western blot detection of phosphoserine/threonine and total lamin A/C with lamin A/C protein immunoprecipitated from RAW264.7 cells that were exposed to iL3 for 0, 5, and 15 min, respectively. Cells were either pretreated with 1, 5, or 10 μM PKCζ pseudosubstrate inhibitor ZIP for 30 min. The PVDF membrane was first probed with an anti-pan phosphoserine/threonine antibody, stripped, and subsequently reprobed with an anti-lamin A antibody to confirm target protein enrichment. **(G)** Quantitative analysis of MET release in RAW264.7 macrophages that were stimulated without or with iL3 for 3 h in the absence or presence of ZIP at indicated concentrations. Data are presented as mean ± SEM (n=6 biological replicates for panel **D**; n=3 for panel **G**). One-way ANOVA with Turkey’s multiple comparisons test **(D)** or with Dunnett’s multiple comparisons test **(G)** was performed for statistical analysis. *P < 0.05, **P < 0.01, ***P < 0.001.

Immunofluorescence imaging confirmed the integrity of the nuclear envelope because the lamin A/C did not rupture upon larvae stimulation in both peritoneal and RAW264.7 macrophages (**Figure 8A**). In addition, membrane-bound DNA vesicles with intact lamin A/C layer demonstrated that the vesicles were derived from the nucleus (**Figure 8A**). Furthermore, we observed the tight apposition of DNA adjacent to the lamin layer in the vesicles’ cortical area (**Figures 8A, B**), resembling the interaction between chromatin and nuclear lamina through lamin-associated domains (LADs) and heterochromatin. We found a progressive increase of lamin A/C phosphorylation upon larval stimulation (**Figure 8C**). To elucidate the involvement of lamin A/C phosphorylation in the nuclear vesicle budding and MET formation, RAW264.7 macrophages were transfected with lentiviral vectors to overexpress the wild-type lamin A/C (WT). Similar transfections were conducted with the mutant lamin A/C carrying single substitution at S423 by alanine (S423A) or aspartic acid (S423D) (**Supplementary Figure 13**). Notably, overexpression of lamin A/C significantly decreased the DNA release compared with the RAW264.7 cells transfected with empty lentiviral vector (**Figure 8D**). In contrast to the S423A and WT, overexpression of S423D mutant resulted in a significantly higher level of DNA discharge upon iL3 stimulation (**Figure 8D**).

Our final experiments sought to discover the kinase responsible for phosphorylation of lamin A/C at S423. Protein kinase C (PKCs) were present in all outputs derived from several kinase prediction platforms (**Supplementary Figure 12B**). Our initial data suggested that PMA, a potent agonist of conventional PKCs and novel PKCs, could not induce MET release (Appendix **Figure S8**) and that Ca^2+^ chelation did not reduce MET production (**Supplementary Figure 4E**). Considering PKCs’ different sensitivity to PMA and dependence on Ca^2+^ for activation (65), we reasoned that atypical PKCs act as the primary kinases catalyzing lamin A/C phosphorylation. As predicted, a gradual accumulation of PKC*ζ* (an atypical PKC) co-immunoprecipitated with lamin A/C in response to iL3 exposure (**Figure 8E**). The specific pseudosubstrate inhibitor, ZIP, suppressed iL3-induced lamin A/C phosphorylation and DNA release in RAW264.7 macrophages (**Figures 8F, G**). These results demonstrated that PKCζ-mediated lamin A/C phosphorylation leads to nucleoplasmic transport and DNA discharge in macrophages exposed to *S. stercoralis* iL3.

## 4 Discussion

**Figure 9:**
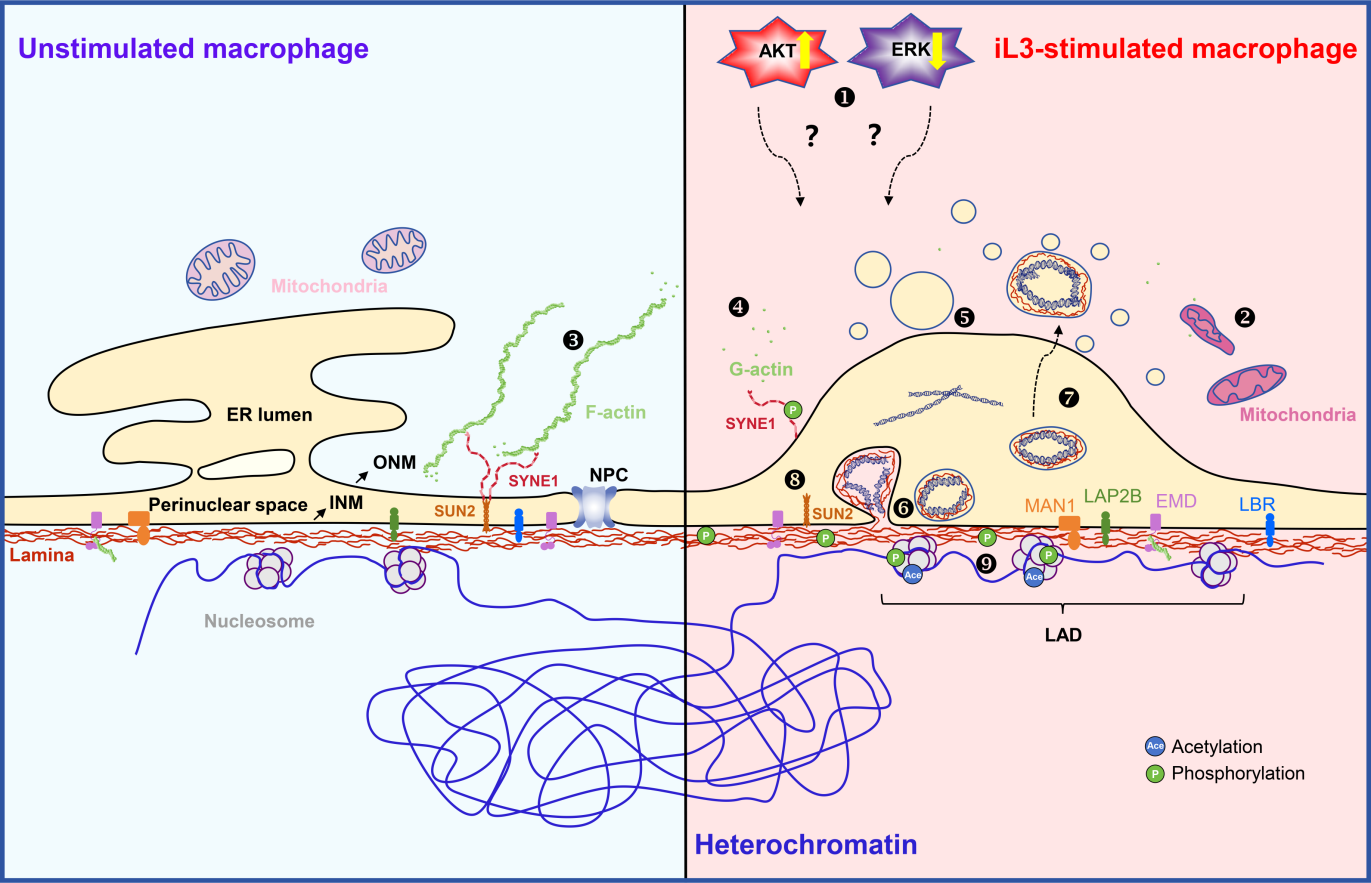
Schematic diagram of the proposed model for cellular mechanisms of MET formation in macrophage driven by *Strongyloides stercoralis* iL3 stimulation. AKT activity is upregulated in macrophages upon *S. stercoralis* iL3 stimulation, thereby positively regulating MET release ❶. Reversely, iL3 induce downregulated activity of ERK. The regulation of ERK and AKT participates in MET formation in an unknown way. The mitochondria show an increased electron density, an elongated or compact appearance, and unclear cristae ❷. The outer nuclear membrane of the nuclear envelope (NE) is a continuous endoplasmic reticulum (ER). Well-organized ER shares a common lumen with the nuclear envelope (ER lumen and perinuclear space). Actin is present in eukaryotic cells in its monomeric globular form (G-actin) or polymerized fibrous form (F-actin) ❸. F-actin is distributed in the perinuclear region in unstimulated macrophages, while disassembles in the perinuclear region in iL3-stimulated macrophages ❹;. Lamin proteins, lamin A/C, lamin B1, and lamin B2, constitute the nuclear lamina by polymerizing and assembling into meshwork underneath the inner nuclear membrane (INM) and in close contact with condensed heterochromatin. Chromatin (mostly heterochromatic) interacts with lamina via the Lamina-associated domain (LAD). Lamins interact with various factors, including nuclear pore complex (NPC) proteins, INM proteins (MAN1, LAP2B, EMD, LBR), chromatin, and chromatin remolding regulators, thereby regulating NE structure. Among INM proteins, EMD (emerin) binding to lamin A/C is required for proper localization to the NE and is predicted to play a role in the genome stabilization and structural rigidity of NE through mediating nuclear actin polymerization underlying the NE. Upon iL3 stimulation, ER disassembles into vesicles and contributes to the increase of outer nuclear membrane (ONM) surface area ❺. Lamina maintains integrity during MET formation ❻. Phosphorylation of lamin A/C facilitates inner nuclear membrane budding and export of chromatin DNA-containing vesicles into the expanded perinuclear space ❼. The physical and functional coupling between the cytoskeleton and the nuclear interior is mainly achieved by the linker of nucleoskeleton and cytoskeleton (LINC) complexes that span the NE. LINC complex comprises Klarsicht, ANC-1, Syne homology (KASH), Sad1, and UNC-84 (SUN) proteins that span the outer and inner nuclear membrane. Cytoplasmic extensions of KASH proteins with distinct domains that bind directly or indirectly to cytoskeletal filaments (115). In the perinuclear space, the lumenal region of SUN proteins, such as SUN2, forms a triple helical coiled-coil, which allows their SUN domains to form a trimer globular head. SUN domains bind to KASH peptides through extensive interactions (115, 116). The nucleoplasmic side of the SUN protein mainly binds to lamin A/C to anchor the LINC complex on the NE (115). Therefore, phosphorylation of SUN2 and SYNE1 could modulate their conformation, anchoring on the nuclear membrane, and interacting with other NE proteins ❽. Even though the interaction of SUN2 and SYNE1 within the perinuclear space partially regulates the distance of the perinuclear space, the dramatically dilated perinuclear space could probably be associated with the disrupted interaction of SUN2 and SYNE1. EMD functions together with lamin A/C in nucleoplasmic anchoring of the LINC complex. The stability and self-assembly of EMD are speculated to be modulated by phosphorylation (117). Forces provided by cytoskeletons acting on the nucleus also lead to local unfolding, conformational changes, and increased phosphorylation of lamins. In addition, the LINC complex, cooperating with lamins and other NE proteins, regulates genome architecture. The negatively charged DNA double helix is complexed with histones, which are positively charged proteins, to form tight nucleosomes. Post-translational modifications such as acetylation and phosphorylation regulate local nucleosome conformation and chromatin condensation ❾.

This study demonstrates that murine macrophages rapidly extrude METs through a non-lytic mechanism upon *S. stercoralis* stimulation *in vitro*, thereby addressing a critical knowledge gap in anti-parasitic innate immunity. Although METs exhibit structural and compositional similarities to ETs derived from neutrophils, eosinophils, and other immune cells (66, 67), the mechanisms of their formation exhibit evident distinctions. The release of METs occurs independently of NADPH oxidase-mediated ROS generation, MPO, neutrophil elastase, and Ca^2^—factors critically required for NET formation (68). *Strongyloides*-induced MET formation involves ultrastructural reorganization marked by ER vesiculation, ONM dilation, and INM budding. ERK/AKT signaling-regulated NE remodeling, F-actin cytoskeletal rearrangement, and histone acetylation serve as the key drivers of MET generation. Phosphorylation of lamin A/C by PKCζ induces INM budding (see the schematic diagram in **Figure 9**), further differentiating the mechanisms underlying the formation of METs and NETs.

A previous study showed mouse bone marrow-derived macrophages (BMDMs) fail to release METs upon stimulation with *S. stercoralis* iL3, possibly attributed to the immaturity state of BMDMs (34) and medium supplements, such as serum and serum albumin (69, 70). The current study demonstrates that peritoneal macrophages, which exhibit a higher degree of differentiation (71, 72), and RAW264.7 macrophages release METs against *S. stercoralis* iL3 in a serum-free medium. Moreover, consistent with previous findings that macrophages are incapable of killing *S. stercoralis* alone (18), our *in vitro* model similarly revealed no significant larvicidal activity mediated by METs. This raises the question of whether METs might require synergistic interactions with other host-derived factors to exert antiparasitic effects, a possibility that warrants further investigation. Unlike the lytic NET formations, which typically require over two hours (73), *S. stercoralis*-induced METs are formed rapidly (within 15 min) in a non-lytic manner. Additionally, the terminally differentiated neutrophils do not require *de novo* gene expression to execute NET release by utilizing pre-existing intracellular factors (74). Likewise, macrophages do not rely on gene transcription to accomplish MET release, highlighting the unique and efficient functional modality of macrophages in executing early immune recognition and defense.

Our study revealed dramatic NE remodeling upon iL3 stimulation, featured by ONM expansion and INM budding. The ONM expansion is likely associated with ER vesiculation and loss of ER-ONM continuity, as ER-derived lipids may flow to and remodel nuclear membranes (75). Similar ER vesiculation observed in NET formation has been implicated in facilitating chromatin DNA externalization across the cytoplasm (76). Underlying the INM is the nuclear lamina, a thick filamentous meshwork, which provides structural stability to the nucleus (77). The nuclear lamina is a meshwork composed of type V intermediate filament proteins, known as lamins, with most mammalian cells expressing the four major types: lamin A, lamin C, lamin B1, and lamin B2 (78). Lamin A and lamin C are splicing isoforms encoded by the single *LMNA* gene and are collectively referred to as lamin A/C (79). The phosphorylation of lamins drives the mitotic disassembly of the NE, while their dephosphorylation is a prerequisite for its post-mitotic reconstruction (80, 81). Likewise, the phosphorylation of either lamin A (82) or lamin B (83) orchestrates NET formation by driving the disassembly of the nuclear lamina and the breakdown of the NE. Notably, despite the dramatic morphological changes we observed in the NE, the process did not involve the disintegration of the nuclear lamina or a breakdown of the NE itself, which maintained its integrity. Our findings establish that MET release is driven by PKCζ-mediated phosphorylation of lamin A/C at a specific residue, Ser423. Unlike phosphorylation events that trigger disassembly, modification at this novel site induces local INM budding to package chromatin for extrusion, thereby facilitating MET release without nuclear lamina disintegration. This molecular strategy fundamentally diverges from NET formation, where phosphorylation of either lamin A (82) or lamin B (83) drives NE breakdown. Intriguingly, nuclear egress bypassing canonical nucleocytoplasmic transport is present in herpesvirus capsid trafficking via NE budding (84, 85) and ribonucleoprotein (RNP) export in *Drosophila* (86). In line with these reports, our work suggests lamin phosphorylation-driven nuclear budding as a universal paradigm complementing the nuclear pore complex (NPC)-mediated transport. Beyond lamins, we identified other phosphorylation events in NPC components and INM proteins, including (1) LEM-domain proteins (LAP2 (lamina-associated polypeptide 2), EMD, MAN1) interacting with lamina and to regulate NE structure (87, 88) (2); members of the linker of nucleoskeleton and cytoskeleton (LINC) complex (SUN2 and SYNE1) forming physical connections in the perinuclear space to transmit forces from cytoskeleton directly to the interior of the nucleus (89). Indeed, perinuclear F-actin disassembly was observed, suggesting the cytoskeleton rearrangement facilitates NE deformation during MET formation.

While NE deformation creates a potential conduit for DNA extrusion, it remains mechanistically perplexing how condensed chromatin could be packaged into INM-derived vesicles given that nuclear DNA is compactly organized into nucleosomes (90). NET formation involves global chromatin decondensation mediated by histone post-translational modifications (PTMs), including citrullination (91, 92), acetylation (93), and methylation (94). This chromatin decondensation provides entropic swelling forces that disrupt NE integrity through mechanical expansion, enabling chromatin extrusion into the extracellular space (95). Notably, histone citrullination is mediated by peptidyl arginine deiminase (PAD) whose activation requires reactive oxygen species (ROS) and calcium influx (42). Thus, our data implicate that histone citrullination is not required for MET formation. Despite the absence of global chromatin decondensation in macrophages undergoing MET formation, histone acetylation was found to promote MET release. Additionally, chromatin decondensation during NET formation requires RNA polymerase-dependent promoter DNA unwinding and transcription activation (96). Thus, our findings lead us to propose that transcription and acetylation-mediated local chromatin conformation modulations may enable chromatin extrusion without large-scale nuclear decompaction. Collectively, given the intricate physical/functional coupling of the cytoskeleton, NE proteins, chromatin, and other nuclear structures (97–100), we propose that MET-associated nuclear deformation is orchestrated by the highly coordinated processes, including cytoskeletal reorganization, NE protein interactions and conformation, and chromatin remodeling (**Figure 9**).

Finally, we demonstrated that ERK and AKT play central roles in signal transduction, regulating cytoskeletal dynamics, endomembrane system organization, and chromatin remodeling (**Figure 9**). These findings align with previous reports documenting the involvement of ERK and AKT in cytoskeletal modulation (55, 101), epigenetic modifications, and gene expression regulation (102, 103). For instance, ERK could modulate histone acetylation through the direct phosphorylation of histone deacetylases (HDACs) (104, 105) and specific chromatin remodeling factors (106). Evidence suggests that AKT signaling regulates acetylation via downregulating the expression of HDACs (107). Besides, through the phosphorylation and activation of ATP-citrate lyase, activated AKT boosts the cellular pool of acetyl-CoA, leading to enhanced histone acetylation (108). Nevertheless, the specific mechanisms through which ERK and AKT coordinate these processes demand further investigation. In contrast to the dependency on ERK activity in NET formation (109–112), this study demonstrated that MET formation is associated with the down-regulation of ERK activity. AKT is essential for NET formation (112). As a critical regulator of apoptosis inhibition, AKT suppression leads to NET formation inhibition via apoptosis induction (113). Likewise, we demonstrated AKT’s central role in regulating cell death pathways, including apoptosis and autophagy, with AKT activity inhibition indeed significantly suppressing MET release. Future studies are required to elucidate how AKT and ERK signaling regulate subcellular events during MET formation.

This study reveals the unique mechanism underlying the rapid release of METs by murine macrophages upon *S. stercoralis* iL3 stimulation and provides novel insights into anti-helminth immune defense. However, the current investigation primarily relies on *in vitro* models, and it remains unclear whether *S. stercoralis* can induce tissue-resident macrophages in diverse tissues to release METs *in vivo*. Future studies should explore (1) the function and mechanism of METs in combating the pathogens (2), whether MET release and the underlying mechanisms are determined by the species and tissue origin of macrophages, activation state, microenvironment, and stimuli (3), subsequent fate of the macrophages after MET release, such as gene expression reprogramming, and functional reconfiguration (4), the mechanistic interplay between ERK and AKT signaling and downstream effectors in governing MET formation. These research directions will deepen the understanding of the physiological functions of METs and also yield potential therapeutic paradigms targeting METs-associated pathologies (114).

## Data Availability

The datasets supporting the conclusions of this article are included within the article and supplementary information files. Further inquiries can be directed to the corresponding authors. The mass spectrometry data have been deposited in the iProX repository with the accession number PXD064553. The data are publicly accessible at https://www.iprox.cn/page/home.html.

## References

[1] Jourdan PM Lamberton PHL Fenwick A Addiss DG. Soil-transmitted helminth infections. Lancet. (2018) 391:252–65. doi: 10.1016/S0140-6736(17)31930-X, PMID: 28882382

[2] Buonfrate D Bisanzio D Giorli G Odermatt P Furst T Greenaway C. The global prevalence of *Strongyloides stercoralis* infection. Pathogens. (2020) 9:468. doi: 10.3390/pathogens9060468, PMID: 32545787 PMC7349647

[3] Nutman TB. Human infection with *Strongyloides stercoralis* and other related *Strongyloides* species. Parasitology. (2017) 144:263–73. doi: 10.1017/S0031182016000834, PMID: 27181117 PMC5563389

[4] Page W Judd JA Bradbury RS. The unique life cycle of *Strongyloides stercoralis* and implications for public health action. Trop Med Infect Dis. (2018) 3:53. doi: 10.3390/tropicalmed3020053, PMID: 30274449 PMC6073624

[5] Lok JB. *Strongyloides stercoralis*: a model for translational research on parasitic nematode biology. WormBook. (2007) 17:1–18. doi: 10.1895/wormbook.1.134.1, PMID: 18050500 PMC3092380

[6] Albarqi MM Stoltzfus JD Pilgrim AA Nolan TJ Wang Z Kliewer SA. Regulation of life cycle checkpoints and developmental activation of infective larvae in *Strongyloides stercoralis* by dafachronic acid. PloS Pathog. (2016) 12:e1005358. doi: 10.1371/journal.ppat.1005358, PMID: 26727267 PMC4703199

[7] Krolewiecki A Nutman TB. Strongyloidiasis: A neglected tropical disease. Infect Dis Clin North Am. (2019) 33:135–51. doi: 10.1016/j.idc.2018.10.006, PMID: 30712758 PMC6367705

[8] Dawkins HJ Grove DI. Attempts to establish infections with *Strongyloides stercoralis* in mice and other laboratory animals. J Helminthol. (1982) 56:23–6. doi: 10.1017/s0022149x00034957, PMID: 6978359

[9] Grove DI Northern C Heenan PJ. *Strongyloides stercoralis* infections in the muscles of mice: a model for investigating the systemic phase of strongyloidiasis. Pathology. (1986) 18:72–6. doi: 10.3109/00313028609090831, PMID: 3725436

[10] Breloer M Abraham D. *Strongyloides* infection in rodents: immune response and immune regulation. Parasitology. (2017) 144:295–315. doi: 10.1017/s0031182016000111, PMID: 26905057

[11] Nolan TJ Bhopale VM SChad GA. *Strongyloides stercoralis*: oral transfer of parasitic adult worms produces infection in mice and infection with subsequent autoinfection in gerbils. Int J Parasitol. (1999) 29:1047–51. doi: 10.1016/s0020-7519(99)00068-5, PMID: 10501615

[12] Abraham D Rotman HL Haberstroh HF Yutanawiboonchai W Brigandi RA Leon O. *Strongyloides stercoralis*: protective immunity to third-stage larvae inBALB/cByJ mice. Exp Parasitol. (1995) 80:297–307. doi: 10.1006/expr.1995.1036, PMID: 7895840

[13] O’Connell AE Hess JA Santiago GA Nolan TJ Lok JB Lee JJ. Major basic protein from eosinophils and myeloperoxidase from neutrophils are required for protective immunity to *Strongyloides stercoralis* in mice. Infect Immun. (2011) 79:2770–8. doi: 10.1128/IAI.00931-10, PMID: 21482685 PMC3191984

[14] Yan L Wang J Cai X Liou YC Shen HM Hao J. Macrophage plasticity: signaling pathways, tissue repair, and regeneration. MedComm (2020). (2024) 5:e658. doi: 10.1002/mco2.658, PMID: 39092292 PMC11292402

[15] Wynn TA Vannella KM. Macrophages in tissue repair, regeneration, and fibrosis. Immunity. (2016) 44:450–62. doi: 10.1016/j.immuni.2016.02.015, PMID: 26982353 PMC4794754

[16] Lechner A Bohnacker S Esser-von Bieren J. Macrophage regulation & function in helminth infection. Semin Immunol. (2021) 53:101526. doi: 10.1016/j.smim.2021.101526, PMID: 34802871

[17] Inclan-Rico JM Siracusa MC. First responders: Innate immunity to helminths. Trends Parasitol. (2018) 34:861–80. doi: 10.1016/j.pt.2018.08.007, PMID: 30177466 PMC6168350

[18] Bonne-Annee S Kerepesi LA Hess JA O’Connell AE Lok JB Nolan TJ. Human and mouse macrophages collaborate with neutrophils to kill larval. Strongyloides stercoralis. Infect Immun. (2013) 81:3346–55. doi: 10.1128/IAI.00625-13, PMID: 23798541 PMC3754234

[19] Nija RJ Sanju S Sidharthan N Mony U. Extracellular trap by blood cells: clinical implications. Tissue Eng Regener Med. (2020) 17:141–53. doi: 10.1007/s13770-020-00241-z, PMID: 32114678 PMC7105514

[20] Munoz-Caro T Rubio RM Silva LM Magdowski G Gartner U McNeilly TN. Leucocyte-derived extracellular trap formation significantly contributes to Haemonchus contortus larval entrapment. Parasit Vectors. (2015) 8:607. doi: 10.1186/s13071-015-1219-1, PMID: 26610335 PMC4661960

[21] McCoy CJ Reaves BJ Giguere S Coates R Rada B Wolstenholme AJ. Human leukocytes Kill *Brugia malayi* microfilariae independently of DNA-based extracellular trap release. PloS Negl Trop Dis. (2017) 11:e0005279. doi: 10.1371/journal.pntd.0005279, PMID: 28045905 PMC5234842

[22] Munoz-Caro T Conejeros I Zhou E Pikhovych A Gartner U Hermosilla C. *Dirofilaria immitis* microfilariae and third-stage larvae induce canine NETosis resulting in different types of neutrophil extracellular traps. Front Immunol. (2018) 9:968. doi: 10.3389/fimmu.2018.00968, PMID: 29867950 PMC5951940

[23] Ehrens A Rudiger N Heepmann L Linnemann L Hartmann W Hubner MP. Eosinophils and neutrophils eliminate migrating *Strongyloides ratti* larvae at the site of infection in the context of extracellular DNA trap formation. Front Immunol. (2021) 12:715766. doi: 10.3389/fimmu.2021.715766, PMID: 34475874 PMC8406770

[24] Ehrens A Lenz B Neumann AL Giarrizzo S Reichwald JJ Frohberger SJ. *Microfilariae* trigger eosinophil extracellular DNA traps in a dectin-1-dependent manner. Cell Rep. (2021) 34:108621. doi: 10.1016/j.celrep.2020.108621, PMID: 33440150

[25] Chuah C Jones MK Burke ML Owen HC Anthony BJ McManus DP. Spatial and temporal transcriptomics of *Schistosoma japonicum*-induced hepatic granuloma formation reveals novel roles for neutrophils. J Leukoc Biol. (2013) 94:353–65. doi: 10.1189/jlb.1212653, PMID: 23709687

[26] Guo AJ Wang L Meng XL Zhang SH Sheng ZA Wei ZK. Newly excysted juveniles of *Fasciola gigantica* trigger the release of water buffalo neutrophil extracellular traps in *vitro* . Exp Parasitol. (2020) 211:107828. doi: 10.1016/j.exppara.2019.107828, PMID: 31917163

[27] Peixoto R Silva LMR Lopez-Osorio S Zhou E Gartner U Conejeros I. *Fasciola hepatica* induces weak NETosis and low production of intra- and extracellular ROS in exposed bovine polymorphonuclear neutrophils. Dev Comp Immunol. (2021) 114:103787. doi: 10.1016/j.dci.2020.103787, PMID: 32791176

[28] Yildiz K Sursal Simsek N Gurcan IS. Determination of extracellular traps structures from sheep polymorphonuclear leukocytes to *Echinococcus granulosus* protoscoleces. Exp Parasitol. (2022) 239:108283. doi: 10.1016/j.exppara.2022.108283, PMID: 35636497

[29] Omar M Abdelal H. NETosis in parasitic infections: A puzzle that remains unsolved. Int J Mol Sci. (2023) 24:8975. doi: 10.3390/ijms24108975, PMID: 37240321 PMC10218887

[30] Doster RS Rogers LM Gaddy JA Aronoff DM. Macrophage extracellular traps: A scoping review. J Innate Immun. (2018) 10:3–13. doi: 10.1159/000480373, PMID: 28988241 PMC6757166

[31] Wei Z Wang Y Zhang X Wang X Gong P Li J. Bovine macrophage-derived extracellular traps act as early effectors against the abortive parasite. Neospora caninum. Vet Parasitol. (2018) 258:1–7. doi: 10.1016/j.vetpar.2018.06.002, PMID: 30105969

[32] Li L Li X Li G Gong P Zhang X Yang Z. Mouse macrophages capture and kill *Giardia lamblia* by means of releasing extracellular trap. Dev Comp Immunol. (2018) 88:206–12. doi: 10.1016/j.dci.2018.07.024, PMID: 30048699

[33] Ding J Xu N Wang J He Y Wang X Liu M. Plancitoxin-1 mediates extracellular trap evasion by the parasitic helminth. Trichinella spiralis. BMC Biol. (2024) 22:158. doi: 10.1186/s12915-024-01958-2, PMID: 39075478 PMC11287892

[34] Bonne-Annee S Kerepesi LA Hess JA Wesolowski J Paumet F Lok JB. Extracellular traps are associated with human and mouse neutrophil and macrophage mediated killing of larval. Strongyloides stercoralis. Microbes Infect. (2014) 16:502–11. doi: 10.1016/j.micinf.2014.02.012, PMID: 24642003 PMC4076910

[35] Davies JQ Gordon S. Isolation and culture of murine macrophages. Methods Mol Biol. (2005) 290:91–103. doi: 10.1385/1-59259-838-2:091, PMID: 15361657

[36] Gambarotto D Hamel V Guichard P. Ultrastructure expansion microscopy (U-ExM). Methods Cell Biol. (2021) 161:57–81. doi: 10.1016/bs.mcb.2020.05.006, PMID: 33478697

[37] Chou MF Schwartz D. Biological sequence motif discovery using motif-x. Curr Protoc Bioinf. (2011) 13:13.15.11–13.15.24. doi: 10.1002/0471250953.bi1315s35, PMID: 21901740

[38] Galioto AM Hess JA Nolan TJ SChad GA Lee JJ Abraham D. Role of eosinophils and neutrophils in innate and adaptive protective immunity to larval *Strongyloides stercoralis* in mice. Infect Immun. (2006) 74:5730–8. doi: 10.1128/IAI.01958-05, PMID: 16988250 PMC1594891

[39] Schneider M. Collecting resident or thioglycollate-elicited peritoneal macrophages. Methods Mol Biol. (2013) 1031:37–40. doi: 10.1007/978-1-62703-481-4_4, PMID: 23824884

[40] Rios FJ Touyz RM Montezano AC. Isolation and differentiation of murine macrophages. Methods Mol Biol. (2017) 1527:297–309. doi: 10.1007/978-1-4939-6625-7_23, PMID: 28116725

[41] Drab D Santocki M Opydo M Kolaczkowska E. Impact of endogenous and exogenous nitrogen species on macrophage extracellular trap (MET) formation by bone marrow-derived macrophages. Cell Tissue Res. (2023) 394:361–77. doi: 10.1007/s00441-023-03832-z, PMID: 37789240 PMC10638184

[42] Thiam HR Wong SL Wagner DD Waterman CM. Cellular mechanisms of NETosis. Annu Rev Cell Dev Biol. (2020) 36:191–218. doi: 10.1146/annurev-cellbio-020520-111016, PMID: 32663035 PMC8499668

[43] Yousefi S Mihalache C Kozlowski E Schmid I Simon HU. Viable neutrophils release mitochondrial DNA to form neutrophil extracellular traps. Cell Death Differ. (2009) 16:1438–44. doi: 10.1038/cdd.2009.96, PMID: 19609275

[44] Yousefi S Gold JA Andina N Lee JJ Kelly AM Kozlowski E. Catapult-like release of mitochondrial DNA by eosinophils contributes to antibacterial defense. Nat Med. (2008) 14:949–53. doi: 10.1038/nm.1855, PMID: 18690244

[45] Hidalgo A Libby P Soehnlein O Aramburu IV Papayannopoulos V Silvestre-Roig C. Neutrophil extracellular traps: from physiology to pathology. Cardiovasc Res. (2022) 118:2737–53. doi: 10.1093/cvr/cvab329, PMID: 34648022 PMC9586562

[46] Bedard K Krause KH. The NOX family of ROS-generating NADPH oxidases: physiology and pathophysiology. Physiol Rev. (2007) 87:245–313. doi: 10.1152/physrev.00044.2005, PMID: 17237347

[47] Taghavi M Mortaz E Khosravi A Vahedi G Folkerts G Varahram M. Zymosan attenuates melanoma growth progression, increases splenocyte proliferation and induces TLR-2/4 and TNF-alpha expression in mice. J Inflammation (Lond). (2018) 15:5. doi: 10.1186/s12950-018-0182-y, PMID: 29588627 PMC5863857

[48] Kim BS Cho IS Park SY Schuller-Levis G Levis W Park E. Taurine chloramine inhibits NO and TNF-alpha production in zymosan plus interferon-gamma activated RAW 264.7 cells. J Drugs Dermatol. (2011) 10:659–65., PMID: 21637907

[49] Wang P Li B Zhou L Fei E Wang G. The KDEL receptor induces autophagy to promote the clearance of neurodegenerative disease-related proteins. Neuroscience. (2011) 190:43–55. doi: 10.1016/j.neuroscience.2011.06.008, PMID: 21684323

[50] Zheng J Cao Y Yang J Jiang H. UBXD8 mediates mitochondria-associated degradation to restrain apoptosis and mitophagy. EMBO Rep. (2022) 23:e54859. doi: 10.15252/embr.202254859, PMID: 35979733 PMC9535754

[51] Iriondo MN Etxaniz A Varela YR Ballesteros U Hervas JH Montes LR. LC3 subfamily in cardiolipin-mediated mitophagy: a comparison of the LC3A, LC3B and LC3C homologs. Autophagy. (2022) 18:2985–3003. doi: 10.1080/15548627.2022.2062111, PMID: 35414338 PMC9673933

[52] Mosesson MW. The role of fibronectin in monocyte/macrophage function. Prog Clin Biol Res. (1984) 154:155–75., PMID: 6089231

[53] Jain AK Barton MC. Bromodomain histone readers and cancer. J Mol Biol. (2017) 429:2003–10. doi: 10.1016/j.jmb.2016.11.020, PMID: 27890782

[54] Chen M Zhang W Gou Y Xu D Wei Y Liu D. GPS 6.0: an updated server for prediction of kinase-specific phosphorylation sites in proteins. Nucleic Acids Res. (2023) 51:W243–50. doi: 10.1093/nar/gkad383, PMID: 37158278 PMC10320111

[55] Lavoie H Gagnon J Therrien M. ERK signalling: a master regulator of cell behaviour, life and fate. Nat Rev Mol Cell Biol. (2020) 21:607–32. doi: 10.1038/s41580-020-0255-7, PMID: 32576977

[56] Dougherty MK Muller J Ritt DA Zhou M Zhou XZ Copeland TD. Regulation of Raf-1 by direct feedback phosphorylation. Mol Cell. (2005) 17:215–24. doi: 10.1016/j.molcel.2004.11.055, PMID: 15664191

[57] Zhao P Sharir H Kapur A Cowan A Geller EB Adler MW. Targeting of the orphan receptor GPR35 by pamoic acid: a potent activator of extracellular signal-regulated kinase and beta-arrestin2 with antinociceptive activity. Mol Pharmacol. (2010) 78:560–8. doi: 10.1124/mol.110.066746, PMID: 20826425 PMC2981393

[58] Manning BD Toker A. AKT/PKB signaling: navigating the network. Cell. (2017) 169:381–405. doi: 10.1016/j.cell.2017.04.001, PMID: 28431241 PMC5546324

[59] Al-Khouri AM Ma Y Togo SH Williams S Mustelin T. Cooperative phosphorylation of the tumor suppressor phosphatase and tensin homologue (PTEN) by casein kinases and glycogen synthase kinase 3beta. J Biol Chem. (2005) 280:35195–202. doi: 10.1074/jbc.M503045200, PMID: 16107342

[60] Torres J Pulido R. The tumor suppressor PTEN is phosphorylated by the protein kinase CK2 at its C terminus. Implications for PTEN stability to proteasome-mediated degradation. J Biol Chem. (2001) 276:993–8. doi: 10.1074/jbc.M009134200, PMID: 11035045

[61] Hall A. Rho GTPases and the actin cytoskeleton. Science. (1998) 279:509–14. doi: 10.1126/science.279.5350.509, PMID: 9438836

[62] Bement WM Goryachev AB Miller AL von Dassow G. Patterning of the cell cortex by Rho GTPases. Nat Rev Mol Cell Biol. (2024) 25:290–308. doi: 10.1038/s41580-023-00682-z, PMID: 38172611 PMC12706751

[63] Dominguez R Holmes KC. Actin structure and function. Annu Rev Biophys. (2011) 40:169–86. doi: 10.1146/annurev-biophys-042910-155359, PMID: 21314430 PMC3130349

[64] Pawar S Kutay U. The diverse cellular functions of inner nuclear membrane proteins. Cold Spring Harb Perspect Biol. (2021) 13:a040477. doi: 10.1101/cshperspect.a040477, PMID: 33753404 PMC8411953

[65] Steinberg SF. Structural basis of protein kinase C isoform function. Physiol Rev. (2008) 88:1341–78. doi: 10.1152/physrev.00034.2007, PMID: 18923184 PMC2899688

[66] Wang Y Du C Zhang Y Zhu L. Composition and function of neutrophil extracellular traps. Biomolecules. (2024) 14:46. doi: 10.3390/biom14040416, PMID: 38672433 PMC11048602

[67] Mukherjee M Lacy P Ueki S. Eosinophil extracellular traps and inflammatory pathologies-Untangling the web! Front Immunol. (2018) 9:2763. doi: 10.3389/fimmu.2018.02763, PMID: 30534130 PMC6275237

[68] Papayannopoulos V. Neutrophil extracellular traps in immunity and disease. Nat Rev Immunol. (2018) 18:134–47. doi: 10.1038/nri.2017.105, PMID: 28990587

[69] Neubert E Senger-Sander SN Manzke VS Busse J Polo E Scheidmann SEF. Serum and serum albumin inhibit *in vitro* formation of neutrophil extracellular traps (NETs). Front Immunol. (2019) 10:12. doi: 10.3389/fimmu.2019.00012, PMID: 30733715 PMC6354573

[70] Zheng Y Zhu Y Liu X Zheng H Yang Y Lu Y. The screening of albumin as a key serum component in preventing release of neutrophil extracellular traps by selectively inhibiting mitochondrial ROS generation. Can J Physiol Pharmacol. (2021) 99:427–38. doi: 10.1139/cjpp-2019-0670, PMID: 32799676

[71] Hu M Pan Z Yang Y Meng C Geng S You M. Different antigen presentation tendencies of granulocyte-macrophage colony-stimulating factor-induced bone marrow-derived macrophages and peritoneal macrophages. In Vitro Cell Dev Biol Anim. (2012) 48:434–40. doi: 10.1007/s11626-012-9535-7, PMID: 22806973

[72] Zhao YL Tian PX Han F Zheng J Xia XX Xue WJ. Comparison of the characteristics of macrophages derived from murine spleen, peritoneal cavity, and bone marrow. J Zhejiang Univ Sci B. (2017) 18:1055–63. doi: 10.1631/jzus.B1700003, PMID: 29204985 PMC5742288

[73] Tan C Aziz M Wang P. The vitals of NETs. J Leukoc Biol. (2021) 110:797–808. doi: 10.1002/JLB.3RU0620-375R, PMID: 33378572 PMC9059135

[74] Sollberger G Amulic B Zychlinsky A. Neutrophil extracellular trap formation is independent of *de novo* gene expression. PloS One. (2016) 11:e0157454. doi: 10.1371/journal.pone.0157454, PMID: 27310721 PMC4911059

[75] Barger SR Penfield L Bahmanyar S. Coupling lipid synthesis with nuclear envelope remodeling. Trends Biochem Sci. (2022) 47:52–65. doi: 10.1016/j.tibs.2021.08.009, PMID: 34556392 PMC9943564

[76] Thiam HR Wong SL Qiu R Kittisopikul M Vahabikashi A Goldman AE. NETosis proceeds by cytoskeleton and endomembrane disassembly and PAD4-mediated chromatin decondensation and nuclear envelope rupture. Proc Natl Acad Sci U S A. (2020) 117:7326–37. doi: 10.1073/pnas.1909546117, PMID: 32170015 PMC7132277

[77] Stiekema M van Zandvoort M Ramaekers FCS Broers JLV. Structural and mechanical aberrations of the nuclear lamina in disease. Cells. (2020) 9:1884. doi: 10.3390/cells9081884, PMID: 32796718 PMC7464082

[78] Wong X Melendez-Perez AJ Reddy KL. The nuclear lamina. Cold Spring Harb Perspect Biol. (2022) 14:a040113. doi: 10.1101/cshperspect.a040113, PMID: 34400553 PMC8805651

[79] Machiels BM Zorenc AH Endert JM Kuijpers HJ van Eys GJ Ramaekers FC. An alternative splicing product of the lamin A/C gene lacks exon 10. J Biol Chem. (1996) 271:9249–53. doi: 10.1074/jbc.271.16.9249, PMID: 8621584

[80] Liu S Xiong F Dou Z Chu L Yao Y Wang M. Phosphorylation of Lamin A/C regulates the structural integrity of the nuclear envelope. J Biol Chem. (2025) 301:108033. doi: 10.1016/j.jbc.2024.108033, PMID: 39615679 PMC11731451

[81] Liu SY Ikegami K. Nuclear lamin phosphorylation: an emerging role in gene regulation and pathogenesis of laminopathies. Nucleus. (2020) 11:299–314. doi: 10.1080/19491034.2020.1832734, PMID: 33030403 PMC7588210

[82] Amulic B Knackstedt SL Abu Abed U Deigendesch N Harbort CJ Caffrey BE. Cell-cycle proteins control production of neutrophil extracellular traps. Dev Cell. (2017) 43:449–462.e445. doi: 10.1016/j.devcel.2017.10.013, PMID: 29103955

[83] Li Y Li M Weigel B Mall M Werth VP Liu ML. Nuclear envelope rupture and NET formation is driven by PKCalpha-mediated lamin B disassembly. EMBO Rep. (2020) 21:e48779. doi: 10.15252/embr.201948779, PMID: 32537912 PMC7403722

[84] Draganova EB Thorsen MK Heldwein EE. Nuclear egress. Curr Issues Mol Biol. (2021) 41:125–70. doi: 10.21775/cimb.041.125, PMID: 32764158 PMC8253559

[85] Klupp BG Mettenleiter TC. The knowns and unknowns of herpesvirus nuclear egress. Annu Rev Virol. (2023) 10:305–23. doi: 10.1146/annurev-virology-111821-105518, PMID: 37040797

[86] Speese SD Ashley J Jokhi V Nunnari J Barria R Li Y. Nuclear envelope budding enables large ribonucleoprotein particle export during synaptic Wnt signaling. Cell. (2012) 149:832–46. doi: 10.1016/j.cell.2012.03.032, PMID: 22579286 PMC3371233

[87] Holaska JM Kowalski AK Wilson KL. Emerin caps the pointed end of actin filaments: evidence for an actin cortical network at the nuclear inner membrane. PloS Biol. (2004) 2:E231. doi: 10.1371/journal.pbio.0020231, PMID: 15328537 PMC509406

[88] Berk JM Tifft KE Wilson KL. The nuclear envelope LEM-domain protein emerin. Nucleus. (2013) 4:298–314. doi: 10.4161/nucl.25751, PMID: 23873439 PMC3810338

[89] Khilan AA Al-Maslamani NA Horn HF. Cell stretchers and the LINC complex in mechanotransduction. Arch Biochem Biophys. (2021) 702:108829. doi: 10.1016/j.abb.2021.108829, PMID: 33716002

[90] Kobayashi W Kurumizaka H. Structural transition of the nucleosome during chromatin remodeling and transcription. Curr Opin Struct Biol. (2019) 59:107–14. doi: 10.1016/j.sbi.2019.07.011, PMID: 31473439

[91] Lewis HD Liddle J Coote JE Atkinson SJ Barker MD Bax BD. Inhibition of PAD4 activity is sufficient to disrupt mouse and human NET formation. Nat Chem Biol. (2015) 11:189–91. doi: 10.1038/nchembio.1735, PMID: 25622091 PMC4397581

[92] Wang Y Li M Stadler S Correll S Li P Wang D. Histone hypercitrullination mediates chromatin decondensation and neutrophil extracellular trap formation. J Cell Biol. (2009) 184:205–13. doi: 10.1083/jcb.200806072, PMID: 19153223 PMC2654299

[93] Hamam HJ Khan MA Palaniyar N. Histone acetylation promotes neutrophil extracellular trap formation. Biomolecules. (2019) 9:32. doi: 10.3390/biom9010032, PMID: 30669408 PMC6359456

[94] Hamam HJ Palaniyar N. Post-translational modifications in NETosis and NETs-mediated diseases. Biomolecules. (2019) 9:369. doi: 10.3390/biom9080369, PMID: 31416265 PMC6723044

[95] Neubert E Meyer D Rocca F Gunay G Kwaczala-Tessmann A Grandke J. Chromatin swelling drives neutrophil extracellular trap release. Nat Commun. (2018) 9:3767. doi: 10.1038/s41467-018-06263-5, PMID: 30218080 PMC6138659

[96] Khan MA Palaniyar N. Transcriptional firing helps to drive NETosis. Sci Rep. (2017) 7:41749. doi: 10.1038/srep41749, PMID: 28176807 PMC5296899

[97] Kalukula Y Stephens AD Lammerding J Gabriele S. Mechanics and functional consequences of nuclear deformations. Nat Rev Mol Cell Biol. (2022) 23:583–602. doi: 10.1038/s41580-022-00480-z, PMID: 35513718 PMC9902167

[98] King MC. Dynamic regulation of LINC complex composition and function across tissues and contexts. FEBS Lett. (2023) 597:2823–32. doi: 10.1002/1873-3468.14757, PMID: 37846646

[99] Fu Y Jing Z Chen T Xu X Wang X Ren M. Nanotube patterning reduces macrophage inflammatory response via nuclear mechanotransduction. J Nanobiotechnology. (2023) 21:229. doi: 10.1186/s12951-023-01912-4, PMID: 37468894 PMC10354937

[100] Su Y Yin X. The molecular mechanism of macrophages in response to mechanical stress. Ann BioMed Eng. (2025) 53:318–30. doi: 10.1007/s10439-024-03616-8, PMID: 39354279

[101] Hua H Zhang H Chen J Wang J Liu J Jiang Y. Targeting Akt in cancer for precision therapy. J Hematol Oncol. (2021) 14:128. doi: 10.1186/s13045-021-01137-8, PMID: 34419139 PMC8379749

[102] Suganuma T Workman JL. MAP kinases and histone modification. J Mol Cell Biol. (2012) 4:348–50. doi: 10.1093/jmcb/mjs043, PMID: 22831833 PMC6283117

[103] Yang Q Jiang W Hou P. Emerging role of PI3K/AKT in tumor-related epigenetic regulation. Semin Cancer Biol. (2019) 59:112–24. doi: 10.1016/j.semcancer.2019.04.001, PMID: 30951826

[104] Latrasse D Jégu T Li H de Zelicourt A Raynaud C Legras S. MAPK-triggered chromatin reprogramming by histone deacetylase in plant innate immunity. Genome Bio. (2017) 18:131. doi: 10.1186/s13059-017-1261-8, PMID: 28683804 PMC5501531

[105] Chen YJ Wang YN Chang WC. ERK2-mediated C-terminal serine phosphorylation of p300 is vital to the regulation of epidermal growth factor-induced keratin 16 gene expression. J Biol Chem. (2007) 282:27215–28. doi: 10.1074/jbc.M700264200, PMID: 17623675

[106] Esnault C Gualdrini F Horswell S Kelly G Stewart A East P. ERK-induced activation of TCF family of SRF cofactors initiates a chromatin modification cascade associated with transcription. Mol Cell. (2017) 65:1081–1095.e1085. doi: 10.1016/j.molcel.2017.02.005, PMID: 28286024 PMC5364370

[107] Xu Z Jia K Wang H Gao F Zhao S Li F. METTL14-regulated PI3K/Akt signaling pathway via PTEN affects HDAC5-mediated epithelial-mesenchymal transition of renal tubular cells in diabetic kidney disease. Cell Death disease. (2021) 12:32. doi: 10.1038/s41419-020-03312-0, PMID: 33414476 PMC7791055

[108] Das S Morvan F Morozzi G Jourde B Minetti GC Kahle P. ATP citrate lyase regulates myofiber differentiation and increases regeneration by altering histone acetylation. Cell Rep. (2017) 21:3003–11. doi: 10.1016/j.celrep.2017.11.038, PMID: 29241530

[109] Wu C Xu X Shi Y Li F Zhang X Huang Y. Neutrophil extracellular trap formation model induced by monosodium urate and phorbol myristate acetate: Involvement in MAPK signaling pathways. Int J Mol Sci. (2024) 26:143. doi: 10.3390/ijms26010143, PMID: 39796001 PMC11719704

[110] Cao C Yu P Chu C Wang Z Xu W Cheng F. Magnesium hydride attenuates intestinal barrier injury during hemorrhage shock by regulating neutrophil extracellular trap formation via the ROS/MAPK/PAD4 pathway. Int Immunopharmacol. (2024) 130:111688. doi: 10.1016/j.intimp.2024.111688, PMID: 38394886

[111] DeSouza-Vieira T Guimaraes-Costa A Rochael NC Lira MN Nascimento MT Lima-Gomez PS. Neutrophil extracellular traps release induced by *Leishmania*: role of PI3Kgamma, ERK, PI3Ksigma, PKC, and [Ca2+. J Leukoc Biol. (2016) 100:801–10. doi: 10.1189/jlb.4A0615-261RR, PMID: 27154356 PMC5014744

[112] Douda DN Khan MA Grasemann H Palaniyar N. SK3 channel and mitochondrial ROS mediate NADPH oxidase-independent NETosis induced by calcium influx. Proc Natl Acad Sci U S A. (2015) 112:2817–22. doi: 10.1073/pnas.1414055112, PMID: 25730848 PMC4352781

[113] Douda DN Yip L Khan MA Grasemann H Palaniyar N. Akt is essential to induce NADPH-dependent NETosis and to switch the neutrophil death to apoptosis. Blood. (2014) 123:597–600. doi: 10.1182/blood-2013-09-526707, PMID: 24458280

[114] Rasmussen KH Hawkins CL. Role of macrophage extracellular traps in innate immunity and inflammatory disease. Biochem Soc Trans. (2022) 50:21–32. doi: 10.1042/BST20210962, PMID: 35191493

[115] Chang W Worman HJ Gundersen GG. Accessorizing and anchoring the LINC complex for multifunctionality. J Cell Biol. (2015) 208:11–22. doi: 10.1083/jcb.201409047, PMID: 25559183 PMC4284225

[116] Jahed Z Domkam N Ornowski J Yerima G Mofrad MRK. Molecular models of LINC complex assembly at the nuclear envelope. J Cell Sci. (2021) 134:jcs258194. doi: 10.1242/jcs.258194, PMID: 34152389

[117] Samson C Celli F Hendriks K Zinke M Essawy N Herrada I. Emerin self-assembly mechanism: role of the LEM domain. FEBS J. (2017) 284:338–52. doi: 10.1111/febs.13983, PMID: 27960036

