# Supplementary material for: New mechanistic insights into macrophage extracellular trap formation induced by a parasitic nematode, Strongyloides stercoralis
Source: Front Immunol. 2025 Oct 24;16:1636232. doi: 10.3389/fimmu.2025.1636232 (PMC12592133; doi:10.3389/fimmu.2025.1636232)
Supplement: Supplementary file 1 [file Table1.docx]

***Supplementary Material***


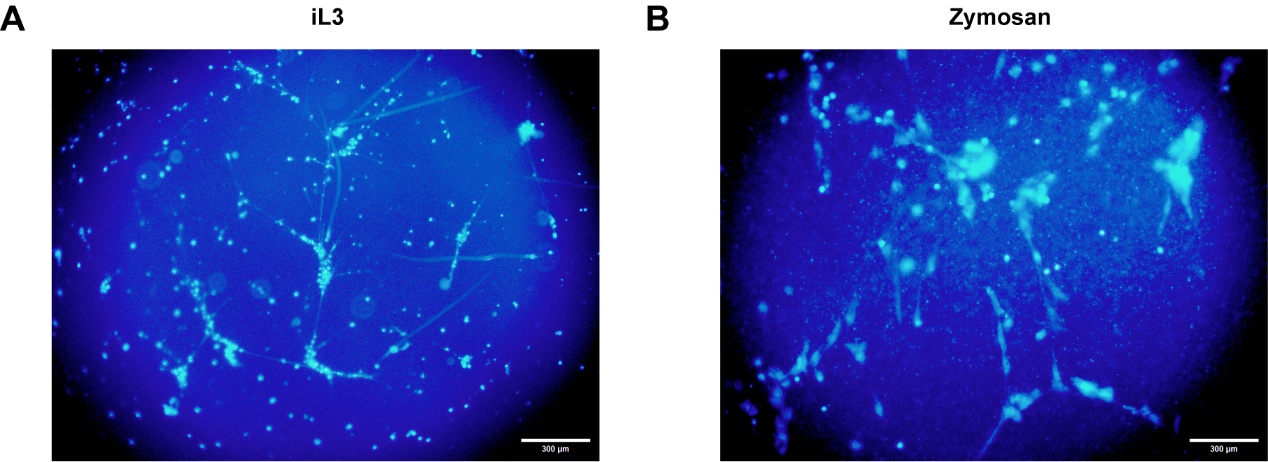


**Supplementary Figure 1.** **Fluorescence images of peritoneal macrophages (PMs) exposed to *Strongyloides stercoralis* iL3 and zymosan.** PMs were exposed to iL3 **(A)** and zymosan **(B)** in the serum-free medium for 3 h in 24-well plates. Hoechst 33258 was added to wells at a final concentration of 5 μg/mL for DNA staining. Cells were observed under the inverted fluorescent microscope. Scale bar=300 μm.


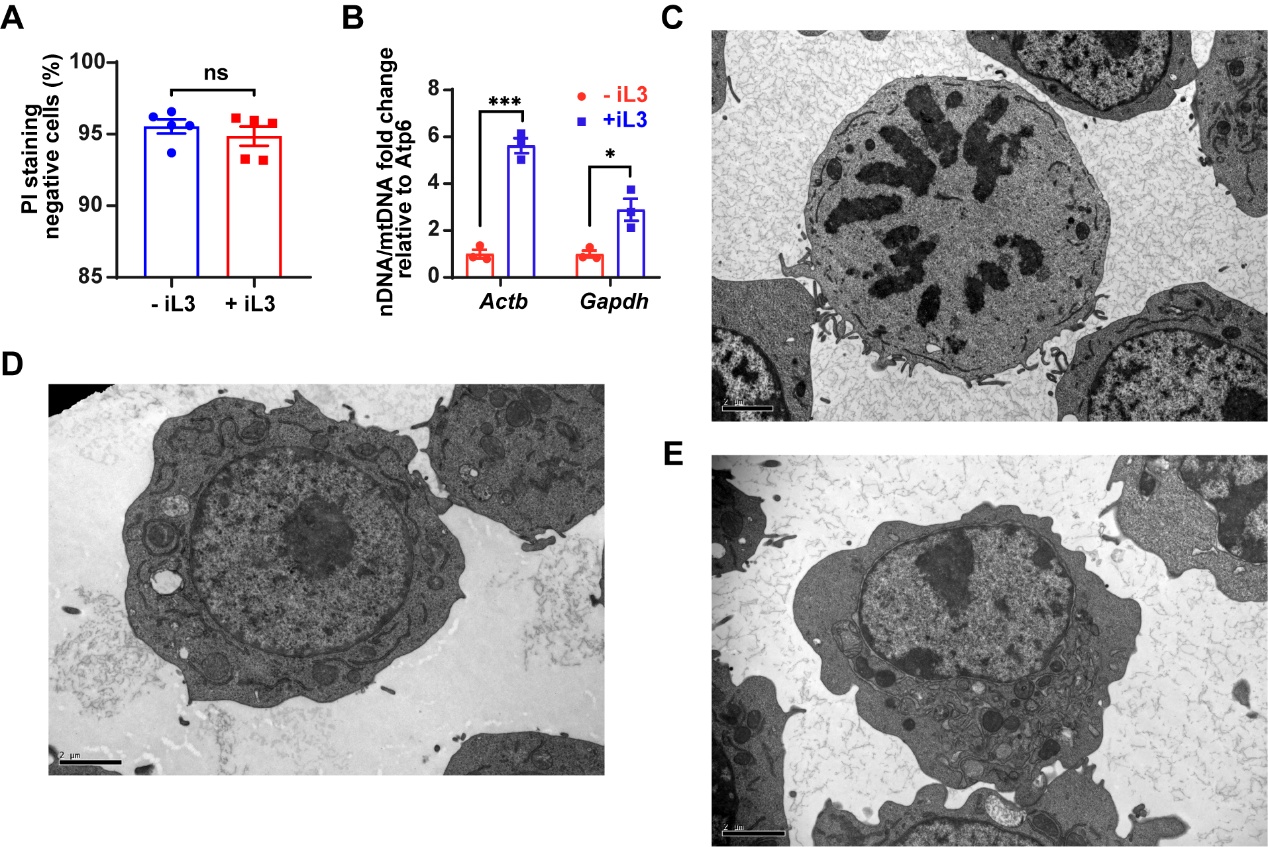


**Supplementary Figure 2. METs are derived from the nucleus with endoplasmic reticulum vacuolation upon *Strongyloides stercoralis* iL3 stimulation. (A)** Percentages of cells negatively stained by propidium iodide (PI). RAW264.7 cells were exposed to *S. stercoralis* iL3 (+iL3) or not(-iL3) for 3 h. Cells were stained with PI for 5 min without fixation. **(B)** Real-time PCR analysis of supernatants from RAW264.7 without iL3 (-iL3) or with iL3 (+iL3) stimulation. Shown is the nuclear DNA (nDNA)/mitochondrial DNA (mtDNA) fold change comparing the nDNA/mtDNA fold change of DNA in supernatants with iL3 stimulation relative to nDNA/mtDNA fold change of those without iL3 stimulation. Histogram shows fold change of two nDNA, *Actb* and *Gapdh*, relative to mtDNA *Atp6*, respectively, upon iL3 stimulation. **(C)** Representative transmission electron microscopy (TEM) image of a RAW264.7 cell undergoing mitosis. Scale bar=2 μm. **(D)** Representative TEM image of RAW264.7 cells without iL3 stimulation. Scale bar=2 μm. **(E)** Representative TEM image of RAW264.7 with iL3 stimulation for 30 min. Scale bar=2 μm. Data are plotted as mean ± SEM (n=5 biological replicates for panel **A**; n=3 for panel **B**) generated from independent experiments. Statistical analysis was performed using two-tailed unpaired t-test. ns, not significant, **P* < 0.05, ****P* < 0.001.


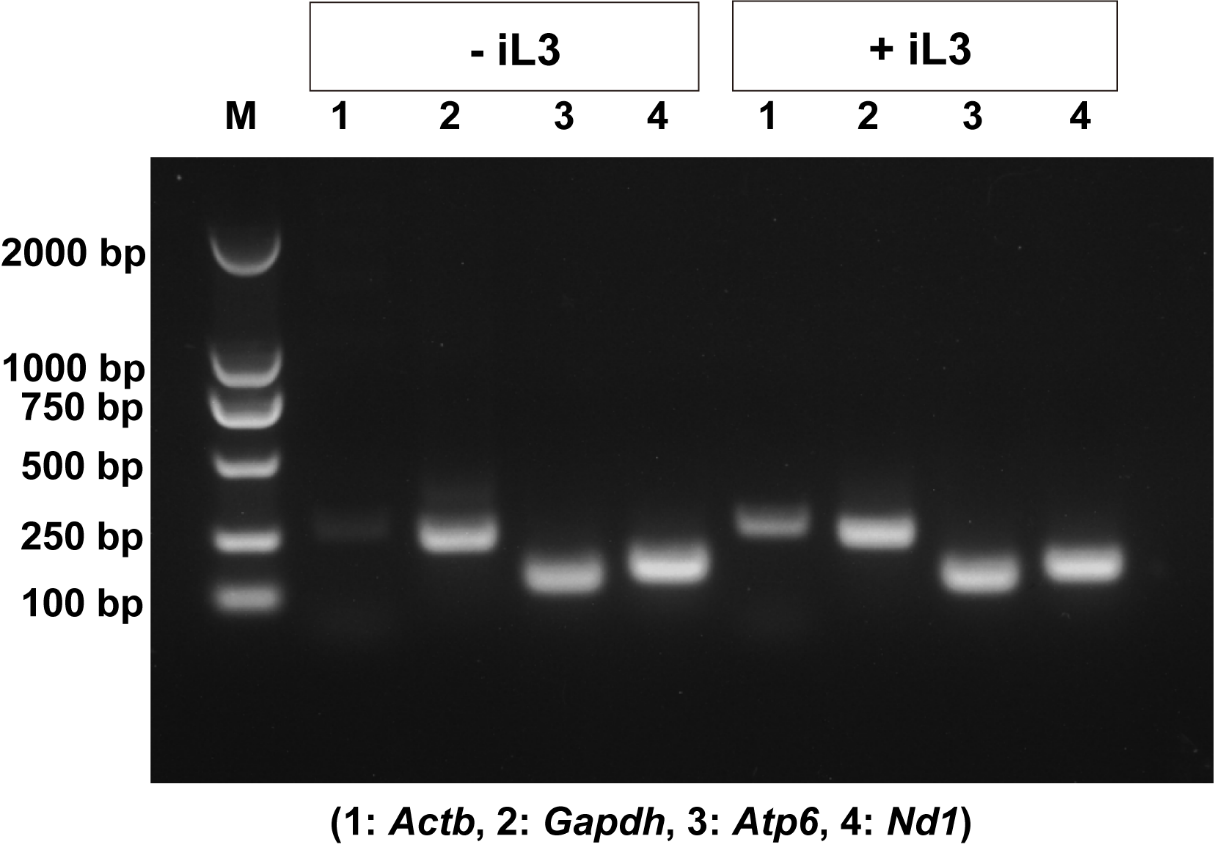


**Supplementary Figure 3. Electrophoretic gel analysis of PCR products of amplified nuclear and mitochondrial genes from supernatants of RAW264.7 exposed to *Strongyloides stercoralis* iL3.** RAW264.7 cells were treated with iL3 (+iL3) or not (-iL3) for 3 h. Total DNA was extracted from supernatants, from which the representative nuclear genes, *Actb*, *Gapdh*, and mitochondrial genes, *Atp6*, *Nd1*, were amplified. PCR products were analyzed by electrophoretic gel.


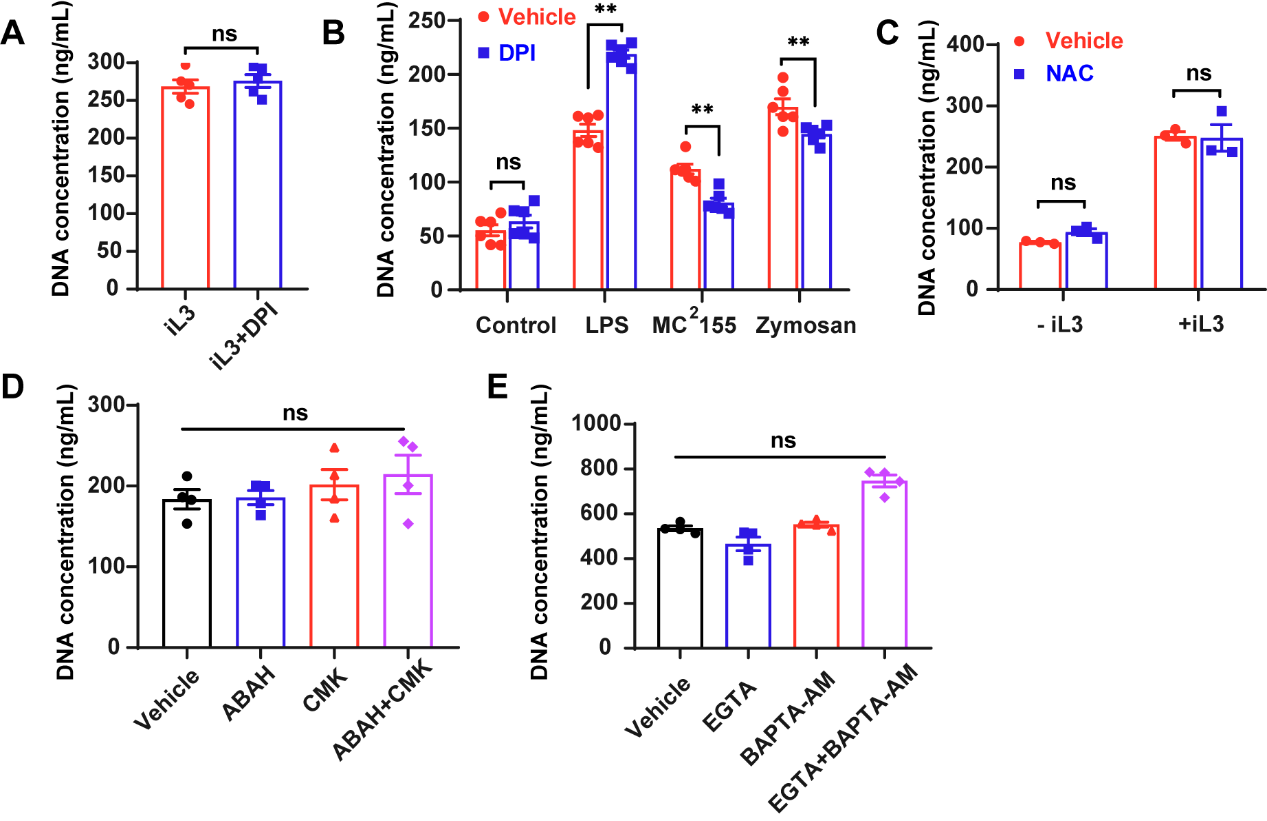


**Supplementary Figure 4. Assessment of the requirement of NADPH oxidase, ROS, myeloperoxidase, neutrophil elastase, and Ca^2+^ for *Strongyloides stercoralis*-induced MET release. (A)** DNA quantification of supernatants from RAW264.7 cells that were pretreated with or without NADPH oxidase inhibitor diphenyleneiodonium chloride (DPI), and were exposed to *S. stercoralis* iL3 for 3 h. **(B)** Quantitative analysis of NADPH oxidase requirement for extracellular DNA release that was induced by different stimuli. RAW264.7 macrophages were pretreated with DPI or with DMSO as vehicle controls, followed by stimulation with LPS, *Mycolicibacterium smegmatis* MC^2^ 155 strain, and zymosan for 3 h. Cell supernatants were collected for DNA concentration determination. **(C)** Quantitative analysis of ROS requirement for MET formation induced by *S. stercoralis*. RAW264.7 macrophages were pretreated with NAC or with DMSO as vehicle controls, followed by stimulation with iL3 for 3h. **(D)** Quantitative analysis of myeloperoxidase (MPO) and neutrophil elastase requirement for *S. stercoralis*-induced MET formation. RAW264.7 macrophages were pretreated with MPO inhibitor 4-Aminobenzohydrazide (ABAH) or neutrophil elastase inhibitor Ac-YVAD-CMK (CMK), respectively or together for 30 min prior to stimulation with iL3 for 3 h. **(E)** Quantitative analysis of Ca^2+^ requirement for *S. stercoralis*-induced MET formation. RAW264.7 macrophages were pretreated without or with cells impermeable (EGTA) and permeable (BAPTA-AM) Ca^2+^ chelators respectively, or together, for 30 min prior to stimulation with iL3 for 3 h. Data are plotted as mean ± SEM (n=5 biological replicates for panels **A**, n=6 for panel **B**; n=3 for panel **C**; n=4 for panels **D** and **E**) generated from independent experiments. Statistical analysis was performed by two-tailed unpaired t-test (**A**, **B**, **C**) and one-way ANOVA with Tukey's multiple comparisons test (**D**, **E**). ns, no statistical significance, ** *P* ≤ 0.01.


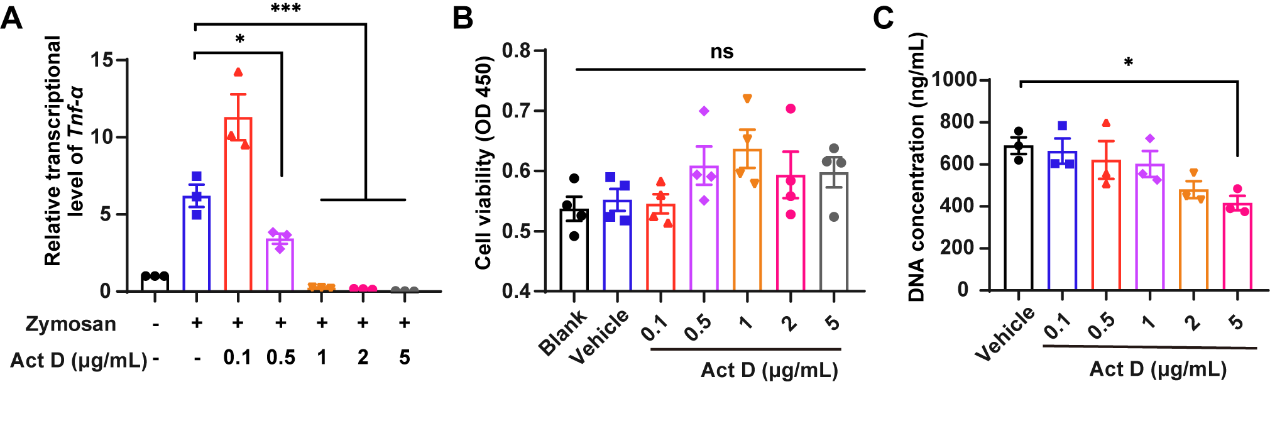


**Supplementary Figure 5. Actinomycin D (Act D) inhibits *Tnf-α* transcription and MET release in *Strongyloides stercoralis* iL3 stimulated RAW264.7 macrophages. (A)** Quantitative real-time PCR (qPCR) analysis of *Tnf-α* gene transcriptional activation in RAW264.7 induced by zymosan and inhibited by Act D in a dose-dependent manner. Cells were pretreated without or with Act D at indicated concentrations for 30 min, followed by exposure to zymosan. Quantitative real-time PCR was performed to determine the relative mRNA level of *Tnf-α*. **(B)** Quantitative analysis of cell viability of RAW264.7 macrophages that were treated with Act D for 3 h in a serum-free medium. Cells treated with DMSO and cells without treatment were set as vehicle control and blank control, respectively. Cells were incubated with CCK-8 reagent for 1 h, absorbance was then measured at 450 nm. **(C)** Quantitative analysis of inhibition of Act D on *S. stercoralis-*induced MET release. RAW264.7 macrophages were pretreated with Act D at indicated concentrations or with DMSO as vehicle control for 30 min, followed by stimulation with *S. stercoralis* iL3 for 3 h. Cell supernatants were collected for DNA quantification. Data are plotted as mean ± SEM (n= 4 biological replicates for panels **A** and **B**; n=3 for panel **C**) generated from independent experiments. Statistical analysis was performed by one-way ANOVA, with Dunnett's multiple comparisons test (**A**, **C**) and Tukey's multiple comparisons test (**B**) . ns, not significant, **P* < 0.05, ****P* < 0.001.


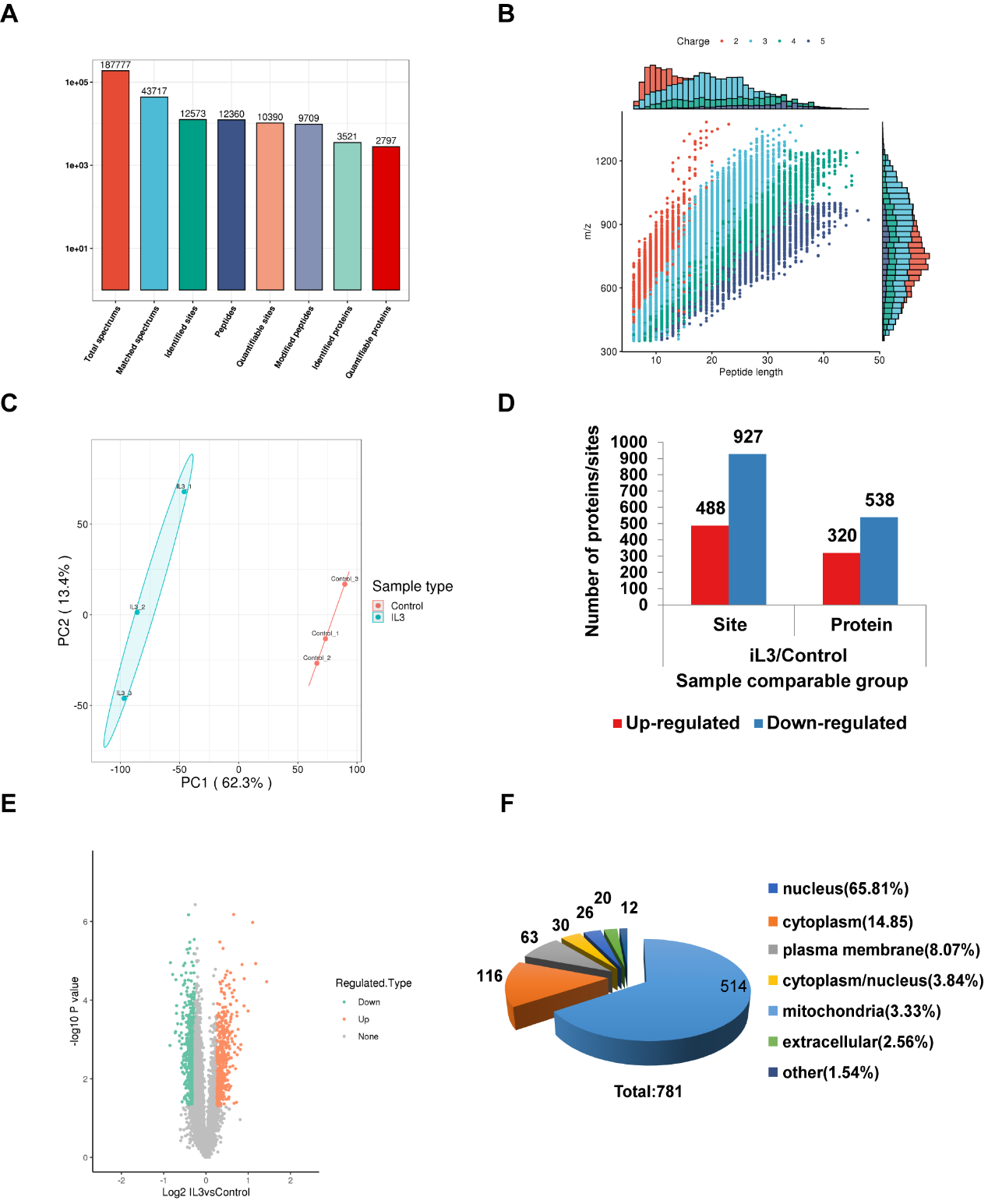


**Supplementary Figure 6. Comparative profiling of differentially modified proteins identified in RAW264.7 macrophages stimulated with *Strongyloides* iL3. (A)** An overview of identified spectrums, sites, peptides, and proteins. Data were filtered by false discovery rate (FDR) ≤ 1%. **(B)** Length distribution of identified peptides. Most of the peptides are distributed in 7-20 amino acids, which conforms to the general rules based on enzymatic hydrolysis and mass spectrometry fragmentation. The distribution of peptide lengths identified by mass spectrometry meets the quality control requirements. **(C)** The protein quantitative principal component analysis (PCA) derived from all samples is shown in the graph, in which the degree of aggregation among samples represents the difference between samples. **(D)** Volcano plot for the comparison of differentially modified sites between *S. stercoralis* iL3-stimulated and non-stimulated RAW264.7 macrophages. Scatters in orange indicate up-regulated sites, while green scatters indicate down-regulated sites. Gray scatters indicate identified sites that were not significantly regulated. **(F)** A summary of identified up-/down-regulated sites and proteins (Fold change ≥ 1.2 or ≤ 0.83, *P* ≤ 0.05 hereinafter inclusive)**. (E)** Subcellular localization of differentially modified proteins.


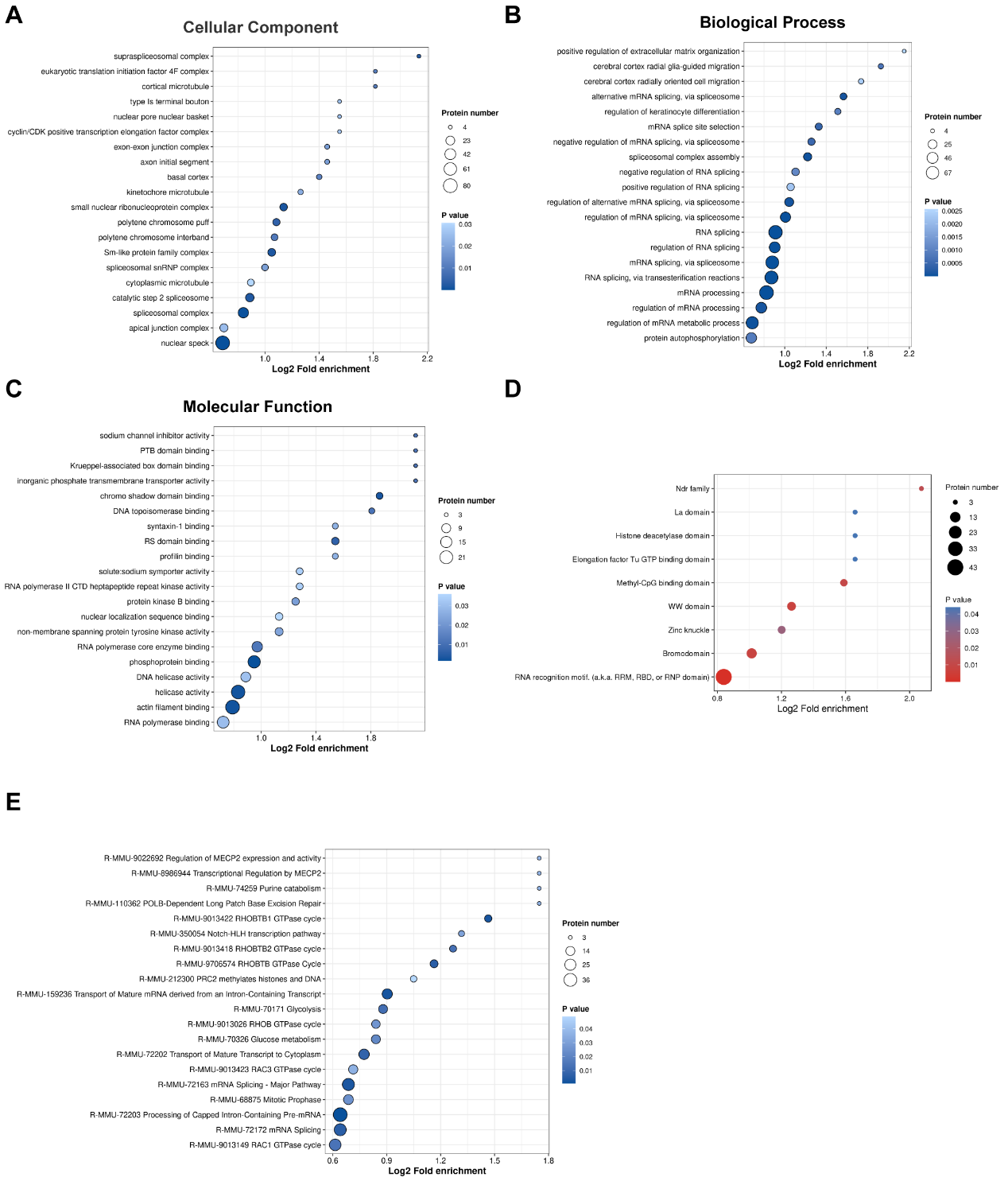


**Supplementary Figure 7. Bioinformatics analysis of differentially modified proteins in *Strongyloides* *stercoralis* iL3-stimulated versus unstimulated RAW264.7 macrophages. (A-C)** Gene ontology (GO) enrichment analysis of differentially modified proteins in *S. stercoralis* iL3-stimulated versus non-stimulated RAW264.7 macrophages. Shown are the top 20 significantly enriched GO terms (*P* ≤ 0.05) in Biological Process **(A)**, Cellular Component **(B)**, and Molecular Function **(C)** categories, respectively. The size of the bubbles indicates the protein number included in the corresponding term. The fold enrichment is shown as a log2 value. The color of the bubbles indicates a corrected *P* value calculated with two-tailed Fisher’s exact test. **(D)** Enrichment analysis of differentially modified proteins (*P* ≤ 0.05). The x-axis shows Log2 Fold enrichment score. The size of the bubbles indicates the protein number included in the corresponding domain. The color of the bubbles indicates a corrected *P* value calculated with two-tailed Fisher’s exact test. **(E)** Pathway enrichment analysis performed using the Reactome analysis tool.


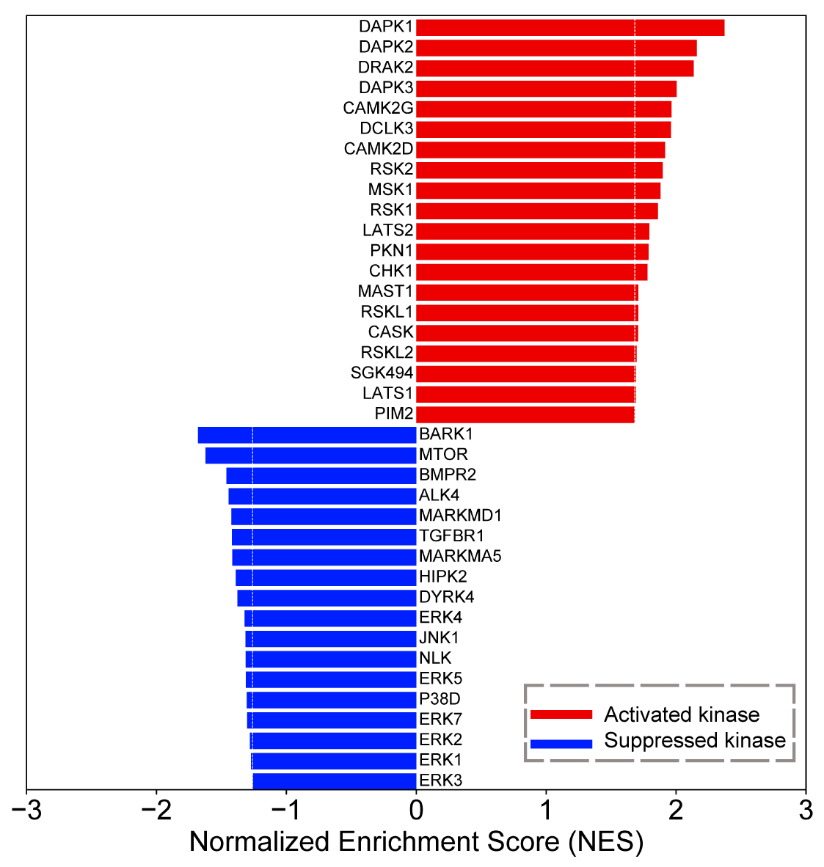


**Supplementary Figure 8. Substrate-based inference of kinase activity changes in *Stongyloides* iL3-stimulated versus unstimulated macrophages.** Kinases responsible for differentially phosphorylated sites were predicted by iGPS 1.0 software. Kinase activity was evaluated by the Gene Set Enrichment Analysis (GSEA) method. The bar chart shows the top 20 activated/suppressed kinases (nominal p < 0.05) in red and blue, respectively. Normalized enrichment score reflects the activation/suppression degree of kinases.


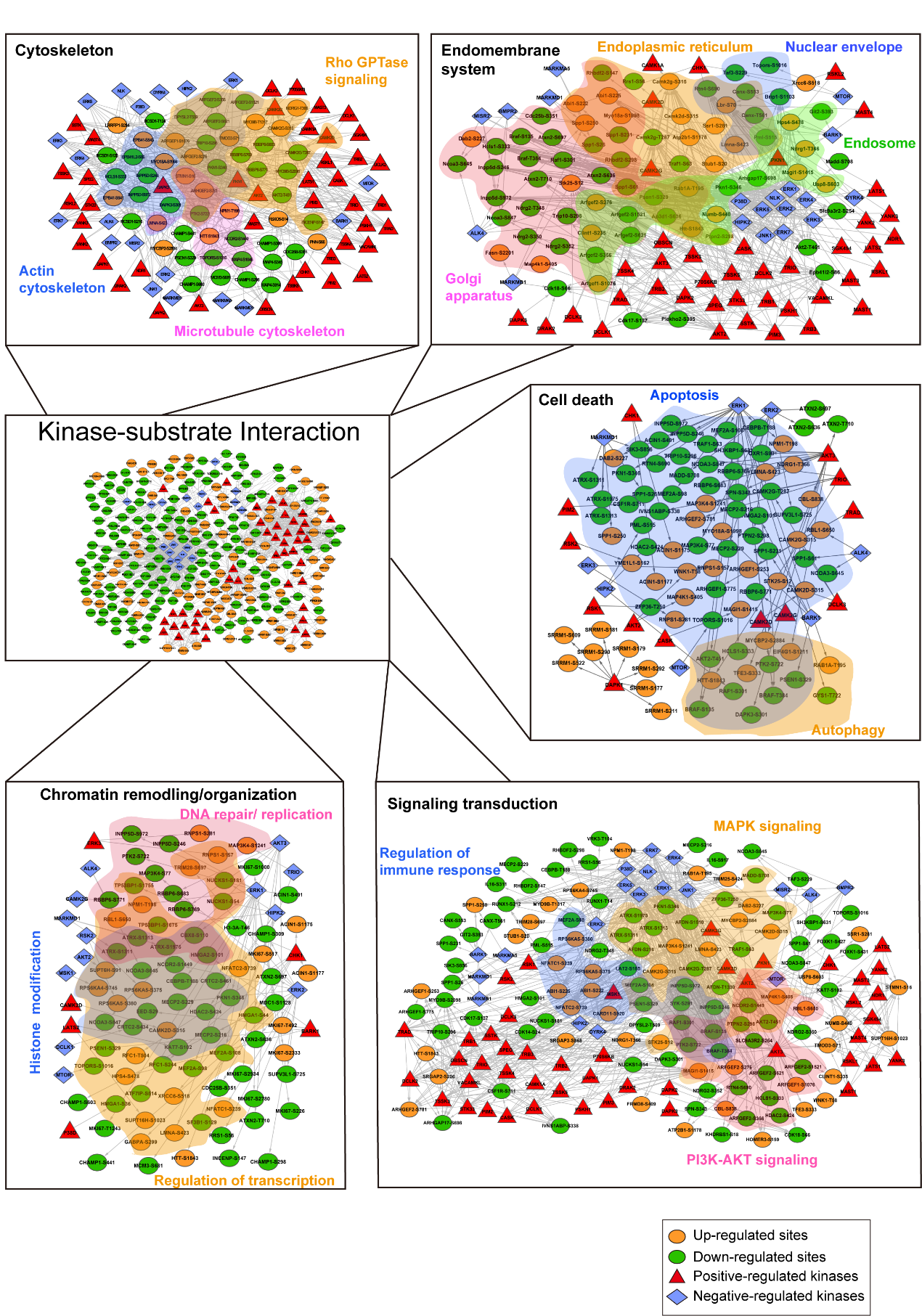


**Supplementary Figure 9. Interaction networks of** **differentially modified substrates and corresponding predicted kinases in *Strongyloides stercoralis* iL3-stimulated versus unstimulated RAW264.7 macrophages.** An interaction network of differentially modified sequence substrates and corresponding predicted kinases was constructed. Substrates were sorted according to GO classification and enrichment. Significantly enriched subsets (*p* < 0.05) of interest were extracted for constructing subnetworks. Different pathways are shown in different color lumps.


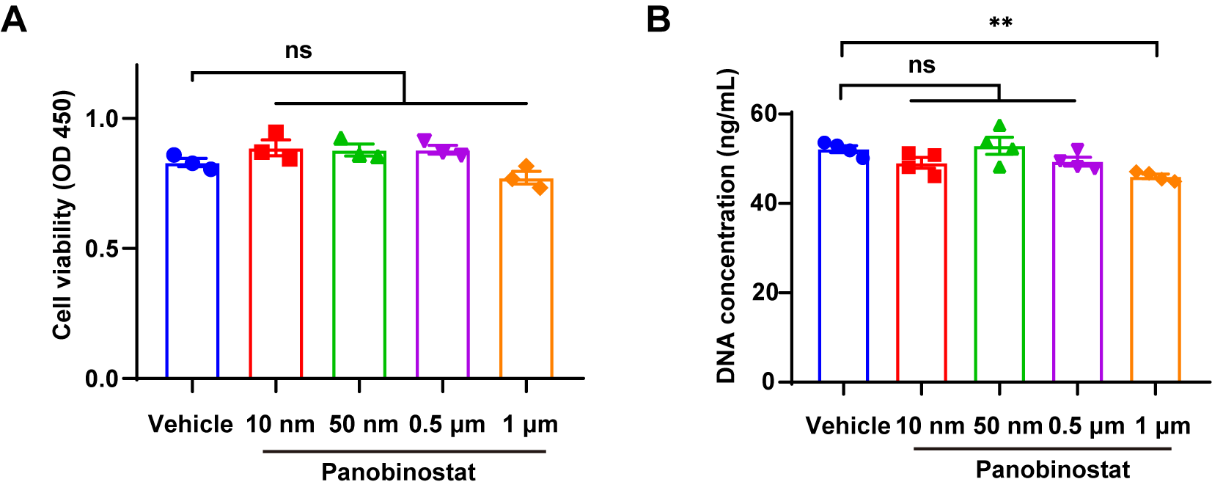


**Supplementary Figure 10. HADC inhibitor panobinostat is not able to affect cell viability and basal DNA release in RAW264.7 macrophages. (A)** Quantitative analysis of cell viability of RAW264.7 macrophages that were treated with panobinostat at indicated concentrations for 3 h in serum-free medium. Cells treated with DMSO were set as vehicle control. Cells were then incubated with CCK-8 reagent for 1 h, and absorbance was then measured at 450 nm. **(B)** Quantitative analysis of DNA concentration of cell supernatants from RAW264.7 macrophages that were treated with panobinostat at indicated concentrations for 3h. Cells treated with DMSO were set as vehicle control. Data are plotted as mean ± SEM (n=3 biological replicates for panel **A**; n=4 for **B**) generated from independent experiments. Statistical analysis was performed by one-way ANOVA with Dunnett's multiple comparisons test. ns, not significant, ***P* < 0.01.


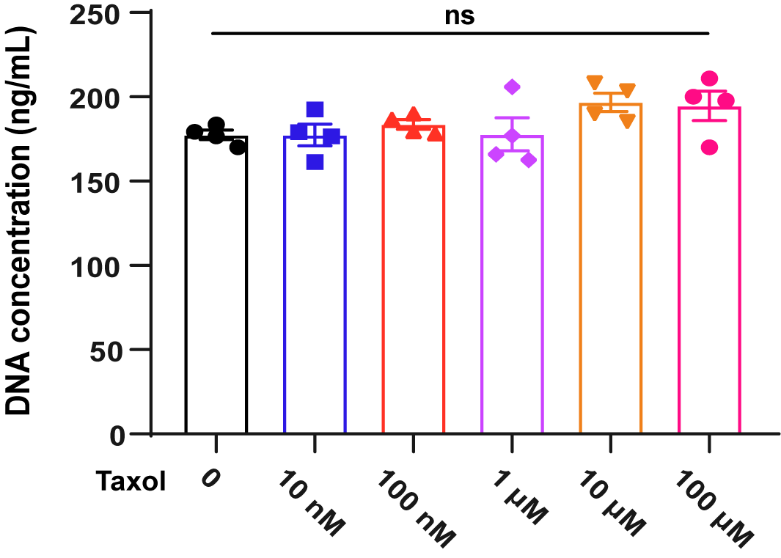


**Supplementary Figure 11. Taxol does not suppress MET release induced by *Strongyloides stercoralis*.** RAW264.7 macrophages were pretreated with taxol at indicated concentrations before *S. stercoralis* iL3 stimulation. Cell supernatants were collected for DNA quantification. Data are plotted as mean ± SEM (n=4 biological replicates) generated from independent experiments. Statistical analysis was performed using One-way ANOVA with Tukey's multiple comparisons test. ns, not significant.


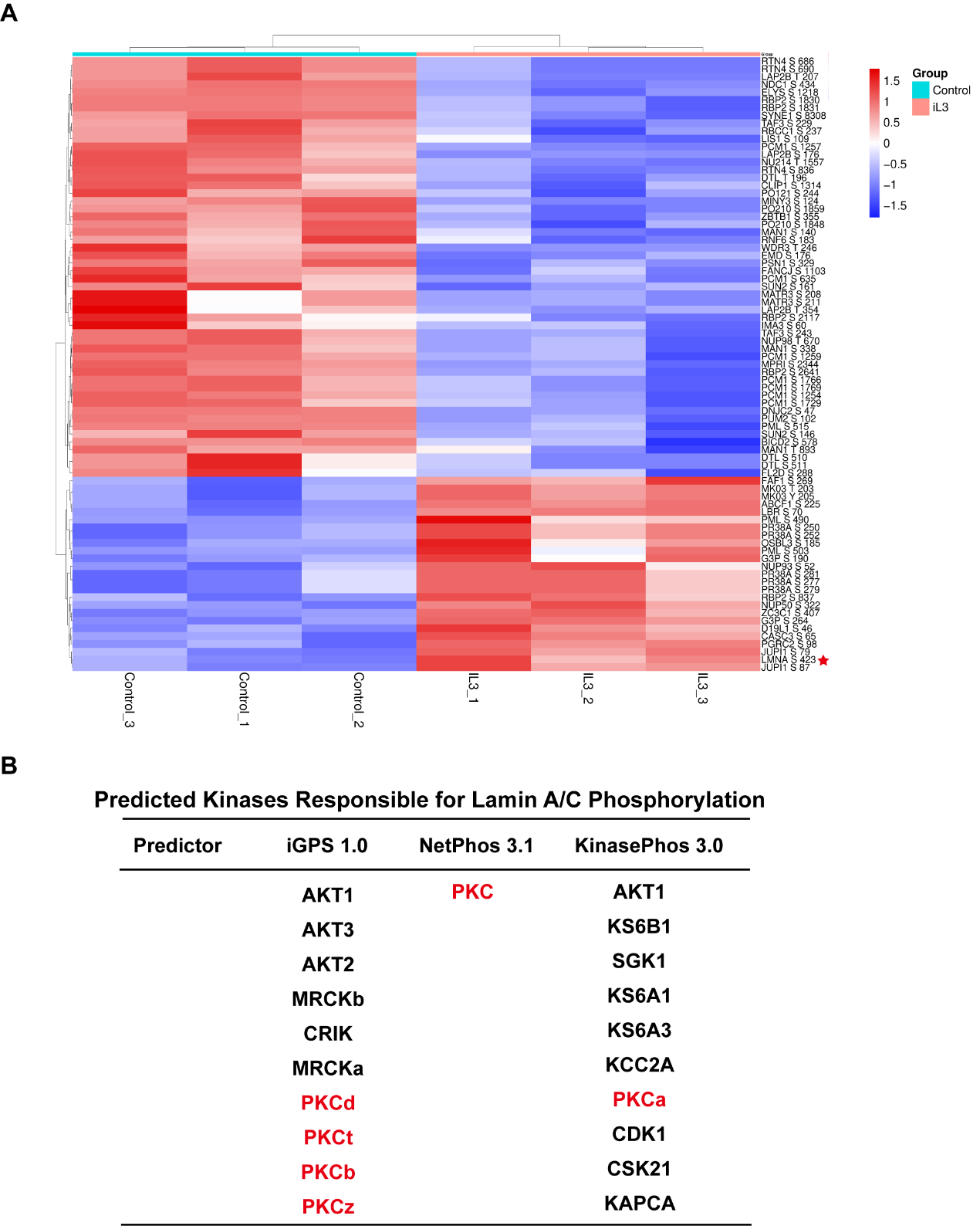


**Supplementary Figure 12. Heatmap of** **nuclear envelope proteins differentially modified in *Strongyloides stercoralis* iL3 stimulated RAW264.7 macrophages versus the unstimulated control, and list of predicted kinase responsible for phosphorylation of lamin A/C. (A)** Heatmap of differentially modified nuclear envelope proteins and corresponding identified sites. Subcellular localization of differentially modified proteins was annotated by the Wolfpsort web tool. Heatmap was generated with the Euclidean clustering distance rows and complete clustering method. Shown are abbreviations of protein names followed by identified modified sites. Lamin A/C (LMNA) is indicated by a red pentacle. **(B)** List of predicted kinases responsible for phosphorylation of lamin A/C at S423 (-KKRKLE**S**SESRSSFS-). Kinases were predicted in iGPS, NetPho 3.1, and KinasePhos 3.0. Protein kinase C isoforms were colored in red.


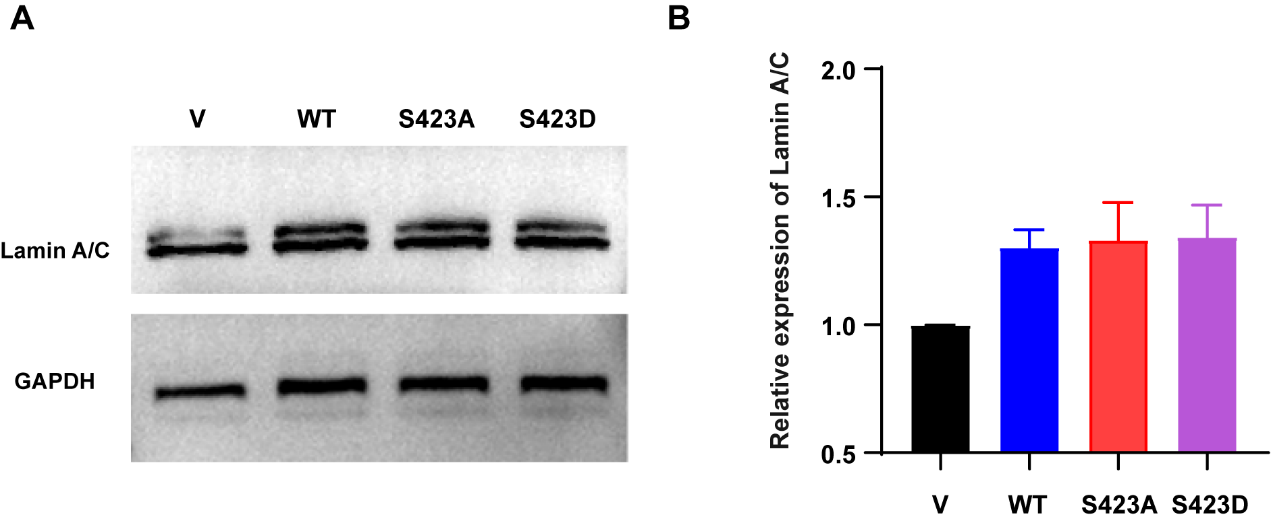


**Supplementary Figure 13. Overexpression of wild-type and phosphorylation-site mutant laminA/C in RAW264.7 macrophages. (A)** Representative western blot of lamin A/C from RAW264,7 macrophages. Cells were transfected with lentiviral vectors containing CDS expressing wild-type form of lamin A/C, mutants that contain single amino acid residue mutation at serine 423 into alanine (S423A) or aspartic acid (S423D). RAW264.7 transfected with lentiviral vectors containing no extra sequence insertion served as empty vector control (V). Cell lysates were analyzed by western blot with an anti-lamin A/C antibody. GAPDH served as a loading control. **(B)** Quantitative analysis of relative expression of lamin A/C. The expression of lamin A/C in each cell was relative to GAPDH and was normalized to empty vector control.


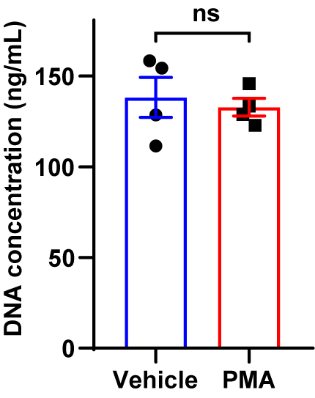


**Supplementary Figure 14. PMA cannot trigger DNA release from RAW264.7 macrophages.** RAW264.7 macrophages in serum-free medium were treated with 100 nM PMA for 3h, cell supernatants were collected for DNA quantification. Data are plotted as mean ± SEM (n=4 biological replicates for panel) generated from independent experiments. Statistical analysis was performed using two-tailed unpaired t-test. ns, not significant.
